# Breeding Chlorophyll-Deficient Mutants of *Chlorella vulgaris* to Enhance Consumer Acceptance

**DOI:** 10.3390/bioengineering13030318

**Published:** 2026-03-10

**Authors:** Malene Lihme Olsen, Daniel Poveda-Huertes, Duygu Ozcelik, Emil Gundersen, Jens Frederik Bang Thøfner, Maryna Kobylynska, Stefania Marcotti, Roland A. Fleck, Damien McGrouther, Johan Andersen-Ranberg, Charlotte Jacobsen, Poul Erik Jensen

**Affiliations:** 1 Department of Food Science, University of Copenhagen, Rolighedsvej 26, 1958 Frederiksberg, Denmark; malene.fog@food.ku.dkduygozcelik@gmail.com (D.O.); jfbt90@gmail.com (J.F.B.T.); 2 Biomass Technology Bioresources Food and Production, Danish Technological Institute, 2630 Taastrup, Denmark; 3 Department of Plant and Environment Sciences, University of Copenhagen, Thorvaldsensvej 40, 1871 Frederiksberg, Denmark; dph@plen.ku.dk (D.P.-H.); joar@plen.ku.dk (J.A.-R.); 4 National Food Institute, Technical University of Denmark, Henrik Dams Allé, 2800 Kongens Lyngby, Denmark; emigu@food.dtu.dk (E.G.); chja@food.dtu.dk (C.J.); 5 Centre for Ultrastructural Imaging, King’s College London, London SE1 1UL, UK; maryna.kobylynska@kcl.ac.uk (M.K.); roland.fleck@kcl.ac.uk (R.A.F.); 6 Randall Centre for Cell and Molecular Biophysics, King’s College London, London SE1 1UL, UK; 7 Crick Advanced Light Microscopy, Francis Crick Institute, London NW1 1AT, UK; stefania.marcotti@kcl.ac.uk; 8 JEOL (UK) Ltd., Welwyn Garden City AL7 1LT, UK; damien.mcgrouther@jeoluk.com

**Keywords:** *Chlorella*, chlorophyll-deficient mutants, microalgae breeding, UV mutagenesis, heterotrophic cultivation, consumer acceptance, non-GMO breeding, microalgae for food, cryo Focused Ion-Beam Scanning Electron Microscopy, vEM, cvEM

## Abstract

The use of microalgae as a food source is limited by consumers’ dislike of their organoleptic traits, primarily the intense green color and bitter taste associated with high chlorophyll content. The eukaryotic microalgae *Chlorella vulgaris* can grow under heterotrophic conditions, providing the opportunity to cultivate chlorophyll-less strains. In this work we applied random mutagenesis for breeding chlorophyll-deficient *C. vulgaris* strains. Wild-type strain was UVC-radiated, and 12 colonies with changed pigmentation were selected. Based on phenotypic stability two mutants, M6 and M11, were selected for characterization of growth, pigment and biomass accumulation. Cultivation under photo-, mixo- and heterotrophic conditions revealed distinct phenotypes for the two mutants. M6 remained chlorophyll-deficient in all cultivation conditions tested, while chlorophyll was observed in M11 when grown under light. Under heterotrophic and mixotrophic growth conditions, both mutants were chlorophyll-deficient while biomass productivity, protein content, and amino acid composition remained similar to wild type. Characterization of the cellular ultrastructure of the wild type and mutants using cryo Focused Ion-Beam Scanning Electron Microscopy revealed that functional chloroplasts and thylakoid membranes were absent in the mutants. Our work demonstrates how a simple approach using UV mutagenesis and visual screening can provide novel strains of *C. vulgaris* with traits for improved consumer acceptance, without compromising the use of the algae biomass as a protein-rich food source.

## 1. Introduction

Eukaryotic microalgae are a large and diverse group of unicellular organisms capable of carrying out photosynthesis, with high growth rates, resulting in a nutritional biomass with the potential to become a sustainable food source that can be cultivated locally [1,2,3,4].

Although microalgae biomass is not completely new as a food source, it is a niche product, mostly sold in health shops as freeze-dried powder or pills for dietary supplements [5,6]. Several factors are limiting consumer acceptance of microalgae in food, including: (1) high cost of production and harvest, (2) often limited digestibility without post-harvest processing and (3) limited organoleptic quality including a “fishy” taste and being considered too green in sensory studies, even when applied in carrier products in small amounts [7,8,9]. Furthermore, consumer studies indicate that European consumers know very little about microalgae: what they are, how they are produced, or how they can be applied in food. Such information is crucial for consumers to accept and integrate novel food items in their diet [10].

*Chlorella vulgaris* is one of the most popular microalgae species approved for food applications [11]. *C. vulgaris* is a green alga with a size in the range of 3–15 µm belonging to the Chlorophyta phylum, from where higher plants have evolved [12]. The algae can grow under phototrophic, mixotrophic and heterotrophic conditions utilizing organic carbon sources such as glucose and acetate [13,14]. The vivid green color of microalgae, often disfavored by consumers, is caused by the presence of chlorophylls, pigments that are key factors in the light harvesting systems of the photosynthetic apparatus and thus essential for carrying out photosynthesis [15,16].

Chlorophyll and its derivatives are potent photosensitizers, meaning that upon illumination they can generate reactive oxygen species (ROS), such as singlet oxygen, which readily attack lipids and other biomolecules. This phenomenon is well established in food systems such as edible oils, where chlorophyll accelerates photooxidation and increases the formation of volatile oxidation products that contribute to off-flavors and reduced oxidative stability [17,18]. Studies on edible microalgae have identified specific volatile organic compounds (VOCs) associated with characteristic sensory attributes described as “seaweed”, “muddy”, or “grassy”. These VOCs largely consist of lipid oxidation products as well as sulfur compounds, aldehydes, ketones, and related metabolites [19]. A particularly relevant study on *Auxenochlorella pyrenoidosa* compared a chlorophyll-deficient mutant with the wild type. The chlorophyll-deficient strain exhibited lower levels of key off-flavor volatiles, including dimethyl trisulfide (rancid/oniony) and nonenal (fishy), resulting in a more neutral and improved odor profile. These findings suggest that reduced chlorophyll content, potentially in combination with associated metabolic changes, can contribute to improved flavor quality [20].

Chlorophylls are non-essential when grown under heterotrophic conditions; thus, the metabolic flexibility of microalgae like *C. vulgaris* facilitates a unique possibility for breeding and cultivating pigment-deficient strains [20,21]. Aside from providing the opportunity to cultivate a pale, chlorophyll-less biomass, the heterotrophic cultivation process entails several other advantages [13]. The challenge of obtaining optimal light utilization in phototrophic cultivation is irrelevant in heterotrophic cultivation and as a result, the biomass productivity for *Chlorella* sp. can be more than 25 times higher than for phototrophic cultivation lowering downstream processing costs per kg biomass substantially [13]. In addition, heterotrophic cultivation offers the opportunity to utilize highly turbid side streams as nutrient media, otherwise limiting phototrophic cultivation [22]. Still, heterotrophic algae cultivation has its drawbacks including an increased risk of bacterial contamination and limited possibility to scale up due to a high oxygen demand which is difficult to meet in highly dense cultures [23]. Heterotrophic cultivation of microalgae is gaining increasing interest in the microalgal industry and is a unique opportunity to produce microalgal biomass with reduced color intensity, neutral taste and smell compared to the green wild type [24,25]. This can in turn increase consumer acceptance by improving the nutritional quality of the carrier food products like pasta and bread, without negatively impacting the color, taste or smell of the final product [8,26]. In this way, heterotrophically cultivated pale algae strains offer consumers a unique opportunity to become more familiar with microalgae as a food source. This familiarity could eventually also pave the way for phototrophic cultivated green algae to be perceived as a more approachable and less unfamiliar food product [27,28].

*C. vulgaris* wildtype cultivated in darkness under heterotrophic conditions has sustained chlorophyll accumulation despite the absence of light [29]. Unlike higher plants, *C. vulgaris* possesses a metabolic pathway for light-independent chlorophyll-a synthesis [30]. Hence, to establish pigment-deficient *C. vulgaris* for heterotrophic cultivation it is necessary to breed chlorophyll deficient strains. This can be achieved by random mutagenesis which in contrast to genetic engineering approaches provides non-GMO strains that have higher consumer acceptance than GMO counterparts [31,32]. Breeding of pigment-deficient strains of *C. vulgaris* using random mutagenesis has been achieved using different approaches including ethyl methane sulphonate (EMS) treatment, UVC-radiation and plasma mutagenesis [24,25,33,34,35]. In this way mutants with up to 90% decrease in pigments and total absence of chlorophyll have been obtained. Compared to traditional food crops that over millennia continue to be improved by breeding, microalgae are a novel food source and therefore hold an inexhaustible potential for strain improvement [36,37].

This study demonstrates how a simple, fast, and cost-effective workflow can serve as an efficient tool—not only for research but also for breeding novel strains more acceptable to consumers. Using the food-approved green microalgae *C. vulgaris* as the model organism, chlorophyll-deficient mutants were generated through random mutagenesis by UVC radiation. Two chlorophyll deficient mutants were selected and characterized with respect to: pigment content, biomass productivity, amino acid and lipid content. To study morphological changes in the mutant cells a newly established method based on cryo Focused Ion Beam Scanning Electron Microscopy (cryo FIB-SEM) was developed and used to give novel insights into changes to the chloroplast ultrastructure of microalgae.

## 2. Materials and Methods

### 2.1. Culture Conditions, Mutagenesis and Mutant Selection

A wild type (WT) strain of *Chlorella vulgaris* (211/11B) (isolated in the Netherlands by M.W. Beijerinck in 1889) from the Culture Collection of Algae and Protozoa (CCAP, Oban, Scotland, UK) was treated with UVC radiation to generate random mutations. *Chlorella vulgaris* (211/11B) was selected for this study due to its status as approved for food consumption. Prior to UVC-radiation, the WT strain was decontaminated as described by Raus et al. [38]. Subsequently the WT was cultivated heterotrophically in 50 mL cell culture flasks containing 30 mL P4-TES media modified from Lippi et al. [39] (See Appendix A for culture media used.). After 5 days of cultivation in darkness on an orbital shaker at 120 rpm at room temperature, the culture was applied for UVC-radiation.

### 2.2. UV Radiation

The heterotrophically cultivated WT *C. vulgaris* culture was diluted to an optical density of 1.0 (OD_750_). A total of 3.0 mL of the suspension was added in a thin layer to empty Petri dishes—just enough to cover the surface—and irradiated at 254 nm for exposure times ranging from 0 to 60 s at an energy intensity of 70,000 µJ/cm^2^, at a distance of 13 cm from the UV-source, using a UVC 500 Ultraviolet Crosslinker (Hoefer, Holliston, MA, USA). To prevent photo-repair by photolyase and to keep the algae in heterotrophic growth mode the plates containing the irradiated algae were placed in darkness for 24 h at 23 °C. After dark incubation, 100 µL each of the UVC-treated cultures including an untreated control were spread on agar plates (modified P4-TES media with glucose as mentioned above incl. 1.2% agar) and placed in the dark at 23 °C until colony-growth appeared after 3–4 weeks. From the solid plates, yellow colonies were isolated by transferring them to new solid plates using sterile toothpicks. Strains were re-streaked on plates to ensure each of them was monoculture.

### 2.3. Screening and Strain Selection

After 3–4 weeks of dark incubation on plates, colonies with a lighter color than the WT were visually selected and re-streaked on new plates (also containing 0.1 M glucose) for incubation with and without light (mixotrophic and heterotrophic cultivation conditions).

### 2.4. Cultivation

Growth of mutants was compared to the WT strain in both photo-, mixo- and heterotrophic growth modes. For mixotrophic and heterotrophic modes, growth was carried out in sterile culture flasks with ventilated caps (Avantor, Radnor, PA, USA) containing 25 mL liquid P4-TES media with 0.1 M glucose, pH 7.5. No glucose was added to the media used for photoautotrophic cultivation. All culture flasks were placed on an orbital shaker at 120 rpm—the ones for photo- and mixotrophic cultivation with constant illumination at 100 µmol m^−2^ s^−1^. The flasks for heterotrophic cultivation were covered in aluminum foil. All cultivations were initiated at an optical density (OD_750_) of 0.2 using precultures in heterotrophic growth for all three strains. Cultivation lasted 7 days, and to avoid contamination, OD_750_ was not measured until the last day.

### 2.5. Dry Matter Determination

A total of 2 mL of each culture was filtered through pre-weighted Macherey-Nagel MN GF-5 glass fiber filters with 0.4 μm pore size and washed with 25 mL demineralized H_2_O. Filters were placed in an oven at 100 °C overnight and weighed again.

The volumetric biomass productivity (dry matter [g]/volume [L]) was determined for the three different stains and cultivations.

### 2.6. Light Microscopy

*C. vulgaris* WT and the chlorophyll deficient mutants cultivated photo-, mixo- and heterotrophically cells from each culture were visually inspected using a light microscope (Olympus BH2, Olympus Danmark, Søborg, Denmark) at 1000× magnification. “ImageJ” (version 1.54f, National Institutes of Health, Bethesda, MD, USA) imaging processing software was used to determine the size of living untreated cells (n: 4–8 cells) and the mean size of the two mutants were compared to the wildtype for each mode of cultivation [40].

### 2.7. Amino Acid Profile

Protein content was measured as the sum of amino acids. The amino acid profile was analyzed in biological triplicates as described by Gundersen et al. (2025) [41] using 15–30 mg dry biomass. Phototrophic cultivations did not produce sufficient amounts of biomass to determine amino acid profile.

### 2.8. Total Lipid

A modified version of the Bligh and Dyer method was applied for total lipid extraction [42,43]. For each sample, 400 mg dried and homogenized biomass was weighed into an extraction glass. Extraction was performed for approximately 4 min by subsequent addition of methanol, chloroform, and water while mixing. To separate the methanol/water, and chloroform/oil phases, samples were centrifuged at 1400× *g* for 10 min. The total lipid content was determined by weighing 15 g of the extract in beakers that were placed in a fume hood for evaporation overnight. Afterwards the lipid content was weighed.

### 2.9. Pigment Analysis

Pigment extraction: A volume of each culture—normalized to 10 OD units at OD_750_—was harvested by centrifugation at 7000 rpm for 5 min. Supernatant was removed, and pigments were extracted from the pellet using 100% acetone. After extraction, acetone was allowed to evaporate, and the pigments were resuspended in 90:10 Metanol:H_2_O containing 5 ppm 8-apocarotenal as internal standard. Pigment extracts were filtered employing a 0.2-μm pore size polyvinylidene fluoride (PVDF) membrane (Agilent, 203980-100, Agilent Technologies Denmark, Glostrup, Denmark) into a 96-well filter plate prior to analysis.

UHPLC-ESI-qTOF-MS analysis: Pigment extracts were analyzed using an Ultimate 3000 UHPLC+ Focused system (Dionex Corporation, Sunnyvale, CA, USA) coupled to a Bruker Compact ESI-QTOF-MS (Bruker, Billerica, MA, USA) system. The analysis method was based on Bijttebier et al. [44]. Samples were separated on a ACQUITY UPLC HSS C18 SB Column, 100 Å, 1.8 µm, 2.1 mm × 100 mm; Phenomenex Inc., Torrance, CA, USA) with a constant temperature of 40 °C and a flow rate of 0.5 mL min^−1^. Samples were injected with a volume of 5 μL. Mobile phase consisted of A: 50:22.5:22.5:5 water + 5 mM ammonium acetate:methanol:acetonitrile:ethyl acetate and B: 50:50 acetonitrile:ethyl acetate. LC gradient: 0–0.1 min, 10% B; 0.1–0.8 min, linear increase from 10 to 30% B; 0.8–20 min, increase 30% to 91% B; 20–20.1 min, increase from 91 to 100% B; 20.1–20.4 min isocratic; 20.4–20.5 min linear decrease from 100 to 10% B; 20.5–23 min isocratic. Pigments were detected by diode array detector (DAD) 350–700 nm. Mass detection was done in positive mode, with a scan range of m/z 100–900 and 2 Hz sample rate. The settings for MS and electrospray ionization (ESI) were: Capillary voltage, 4000 V; end plate offset, 500 V; dry gas temperature, 220 °C; dry gas flow, 8 L min^−1^; nebulizer pressure, 2 bar; in source CID energy, 0 eV; hexapole RF, 50 Vpp; quadrupole ion energy, 4 eV; and collision cell energy, 7 eV.

Raw chromatogram data were calibrated using an internal sodium formate standard. Data analysis was done with DataAnalysis 4.3 (Bruker, Billerica, MA, USA) and Sig-maplot 14 (Systat Software Inc., San Jose, CA, USA). Pigments were quantified by peak area of each pigment measured at UVC absorption at 445 nm. Quantification was done by normalizing to the peak area of 8-apo-carotenal. Relative quantification was normalized to pigment level in the control strain. Identity of all carotenoids mentioned was confirmed by authentic standards.

### 2.10. Cryo Focus Ion Beam Scanning Electron Microscopy for Cryo Volume Electron Microscopy (cvEM)

The WT *C. vulgaris* and the two mutants M6 and M11 in linear, heterotrophic growth were vitrified by pipetting (3 µL) onto TEM grids (Au, 300 R1.2/1.3, Quantifoil, Großlöbichau, Germany), blotted (30 s at 98% RH) to remove excess media and plunged into liquid ethane (EM GP, Leica Microsystems) at −180 °C causing the cells to vitrify into a glass-like solid meta stable state without formation of ice-crystal [45]. All data were acquired using a JEOL JIB-4700F Z FIB-SEM (JEOL, Akishima, Tokyo, Japan), equipped with a Leica microsystems EM VCT500 cryo stage and cryo transfer system (Leica Microsystems, Wein, Austria). Images were collected as a sequence by sectioning in 50 nm slices by Gallium Focused Ion Beam (FIB) followed by imaging of the newly exposed face of each cell with SEM. Volumes were initially generated following alignment of image stacks (Stacker-Neo and Visualizer-evo, TEMography.com, Tachikawa, Tokyo, Japan).

### 2.11. Quantification of Lipid Volume to Cell Volume

Multiple images were sequentially acquired for each slice. To correct motion artifacts in these time series, a single average projection was obtained, resulting in a single image for each z-slice. This step was performed in ImageJ/Fiji (National Institutes of Health, Bethesda, MD, USA) with the Z-project command [46]. The 3D volume was then registered using a previously described algorithm [47]. Briefly, images are first pre-registered using the Linear Stack Alignment with SIFT Fiji plugin [48] with default settings. Subsequently, the Alignment to Median Smoothed Template (AMST) algorithm is applied to refine registration. Afterwards, the Pixel Classification workflow in Ilastik (https://www.ilastik.org/) was used to segment individual lipid droplets within the microalgal cells [49] ImageJ/Fiji was used to manually segment individual cells to provide context for the analysis of lipid droplets. Three cells for each algae strain were analyzed to quantify the total lipid content per cell volume.

### 2.12. Statistical Analysis

All analyses were conducted in triplicates except for the lipid content measurements that were only made as single determinations due to insufficient sample amounts. Graphs and statistical analysis were made in “GraphPad prism 10” applying one-way analysis of variance (ANOVA) to determine if differences between strains and cultivations were significant.

For the pigment analysis: Statistical comparisons between two groups (mixotrophic and heterotrophic cultivation) were performed using Student’s *t*-test (each pigment from the two mutants was compared pairwise to the reference strain (WT) at each cultivation condition). The data are represented by means +/− standard deviation and the different statistical significances are shown as following: n.s. (not significant), *p* > 0.05, * *p* < 0.05, ** *p* < 0.01, *** *p* < 0.001 and **** *p* < 0.0001.

## 3. Results

### 3.1. UV-Mutagenesis

**Figure 1:**
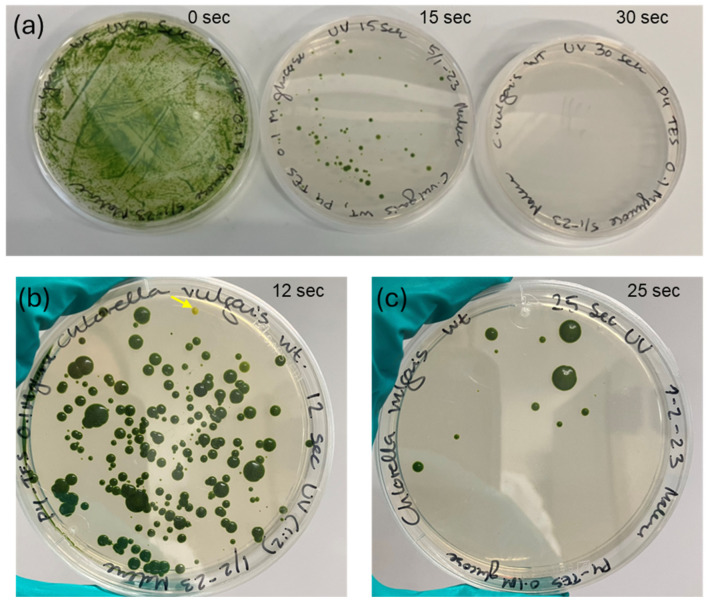
Visualization of agar plates after UV-mutagenesis. (**a**) UVC-radiation dose response illustrated by plating treated and untreated *C. vulgaris* on P4-TES plates with 0.1 M glucose incubated in darkness for 4 weeks. From left plates with 1: the negative control with the un-radiated *C. vulgaris* WT and plate 2; 15 s of radiation and 3; after 30 s UVC-radiation where all cells died. (**b**): from a second independent experiment 12 s of UVC-radiation was an appropriate UVC dose for obtaining plates with separate colonies including pigment mutants (indicated by yellow arrow). (**c**): after 25 s of radiation only a few cells survived.

A wild type strain of *C. vulgaris* was treated with UVC-radiation to induce single nucleotide mutations randomly throughout the genome. After 3–4 weeks of incubation, the plates with cells treated with 12–15 s UVC radiation were the best suited for mutant isolation since colonies were scattered individually including pigment mutants (Figure 1). UVC exposure for 30 s was enough to kill all the cells.

### 3.2. Selection of Pigment-Deficient Mutants

**Figure 2:**
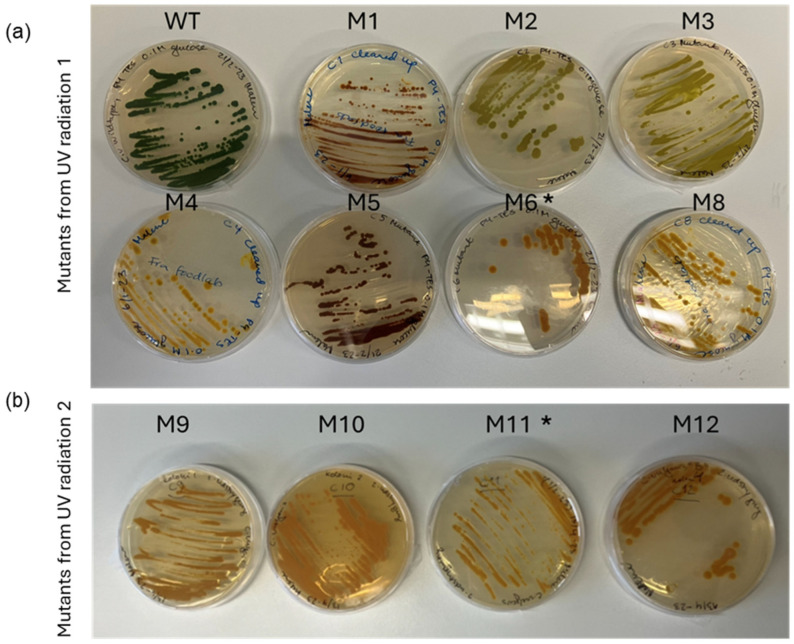
Glucose containing agar plates with the 11 isolated mutants. (**a**) WT and 7 mutants from first radiation trial and (**b**) mutants from the 2nd radiation trial. The two strains selected for further characterization: M6 and M11 are indicated by an asterisk.

To ensure robustness of the procedure the UVC radiation experiment was performed twice with a reproducible outcome, i.e., 12–15 s of UVC radiation resulted in survival of green colonies and a few yellow colonies. From the two UVC experiments, 11 yellow or lime green colonies were selected and re-streaked on fresh plates (Figure 2) and one mutant, M7, had an albino phenotype and showed poor growth. The 12 mutants are described in Table 1. To obtain axenic mutant strains and to test mutation stability of the 11 mutants, they were re-streaked on glucose containing agar plates and incubated in the dark. This was repeated six times. After obtaining clean cultures the phenotypic stability of the mutants was evaluated under both mixotrophic and heterotrophic conditions. Two of the mutants, M6 and M11, did not revert to green during mixotrophic cultivation conditions. These were selected for further characterization.

### 3.3. Cultivations and Biomass Productivity

**Figure 3:**
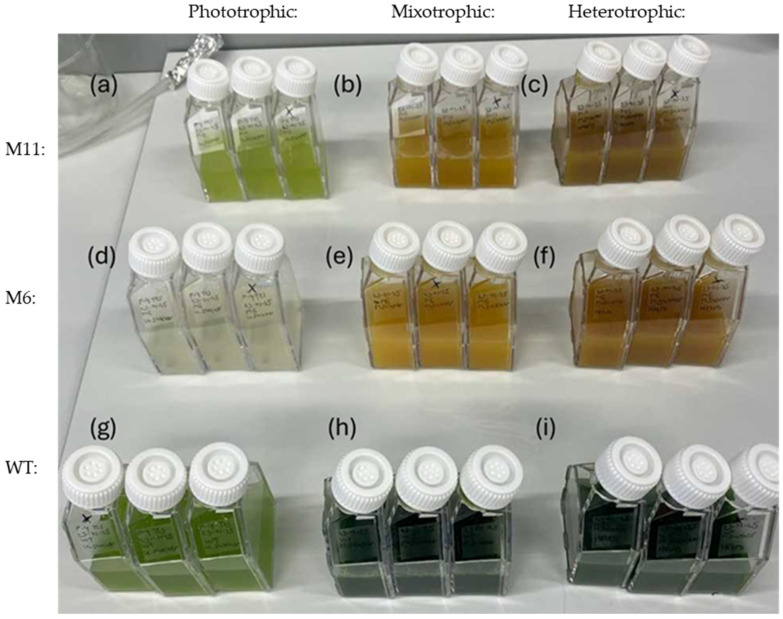
Cell culture flasks after 7 days of cultivation at: phototrophic, mixotrophic and heterotrophic cultivation setups (biological triplicates). (**a**–**c**) Mutant M11: (**a**) phototrophic, (**b**) mixotrophic and (**c**) heterotrophic cultivation (**d**–**f**) Mutant M6: (**d**) phototrophic, (**e**) mixotrophic and (**f**) heterotrophic cultivation (**g**–**i**), *C. vulgaris* WT: (**g**) phototrophic, (**h**) mixotrophic and (**i**) heterotrophic cultivation.

To gather further information about the phenotypic stability of M6 and M11, the two mutants and the WT were cultivated in liquid media at both photo-, mixo- and hetero-trophic conditions. After seven days of cultivation, the biomass density (OD_750_) and dry matter content was measured for all cultivations. As seen in Figure 3, the colors of the cultures varied considerably depending on the type of cultivation.

**Figure 4:**
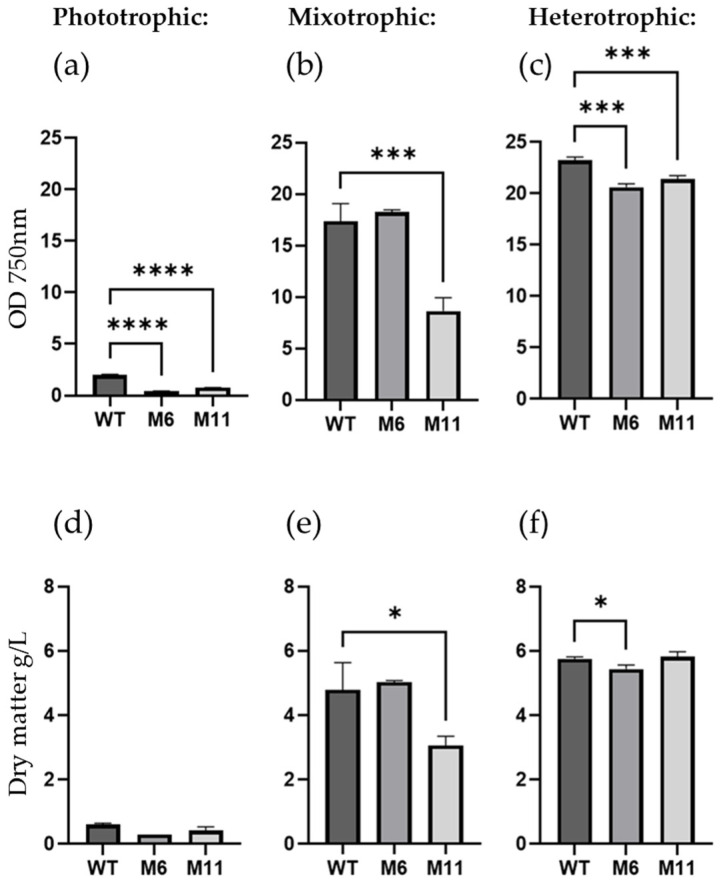
Growth of mutants under photo-, mixo- and heterotrophic conditions as measured by OD_750_ and dry matter content. (**a**–**c**): optical density measured at OD_750_, and (**d**–**f**) dry matter content g/L for *C. vulgaris* (WT) and the two chlorophyll deficient mutants M6 and M11 cultivated both phototrophically (**a**,**d**), mixotrophically (**b**,**e**), and heterotrophically (**c**,**f**). All measurements are made in biological replicates (*n* = 3) after seven days of cultivation. Significant differences are indicated as: * (*p* < 0.05), *** (*p* < 0.005) and **** (*p* < 0.0001).

As expected, the WT strain would grow at all trophic modes with maximum growth observed at heterotrophic conditions and the lowest growth at phototrophic cultivation (Figure 4a). The color of the WT culture was bright green during phototrophic cultivation (Figure 3g) and darker green at both mixotrophic and heterotrophic cultivation (Figure 3h,i).

Based on cell density measured by OD_750_, the WT grew better than both mutants in heterotrophic conditions (Figure 4c). However, in heterotrophic conditions the dry matter content of M11 was similar to WT, with both strains accumulating about 6 g dry matter/L in 7 days, while M6 produced slightly less biomass (Figure 4f). In agreement with the chlorophyll-deficient phenotype, no significant increase in biomass or OD_750_ was observed for M6 in phototrophic cultivation conditions when compared to WT (Figure 4d). The culture density (OD_750_) of M11 increased from 0.2 to 0.7 during the seven days of phototrophic cultivation (Figure 4d) in agreement with the M11 culture turning from yellow to light green under phototrophic cultivation (Figure 3a). Under heterotrophic conditions, M11 doubled the biomass relative to mixotrophic conditions (Figure 4f and Figure 4e, respectively).

### 3.4. Visual Inspection Using Light Microscopy and Cell Size Measurement

**Figure 5:**
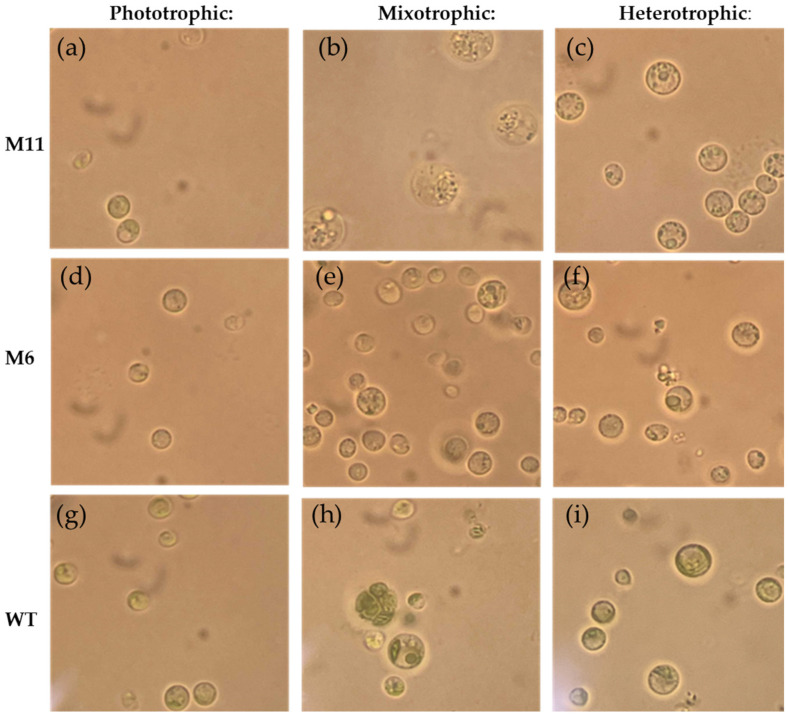
Light microscopy micrographs of mutant M11, M6 and WT strain after 7 days of cultivation at three trophic modes. Mutant M11 cultivated photo- (**a**), mixo- (**b**) and heterotrophically (**c**). Mutant M6 cultivated photo- (**d**), mixo- (**e**) and heterotrophically (**f**). WT strain cultivated at photo- (**g**), mixo- (**h**) and heterotrophic conditions (**i**). All micrographs are at 1000× magnification, scale bar: 10 µm. Color corrected to reduce yellow background. Original micrographs (Figure S4, Supplemental).

Light microscopy of cells from the *C. vulgaris* WT, mutant M6 and M11 revealed that the morphology of all three cultures varied depending on the mode of cultivation (Figure 5). Wild-type strain had visible chloroplasts in all trophic modes (Figure 5g–i), while morphological changes in the chloroplast in M6 and M11 were clearly observed during mixotrophic and heterotrophic cultivation. In phototrophic cultivation of M11 a structure resembling a chloroplast was identified (Figure 5a). Furthermore, in mixotrophic cultivation enlarged cells of M11 appeared “grainier” compared to the phototrophic and heterotrophically cultivated cells. A ring-shaped object resembling the pyrenoid could be identified in WT M6, and M11 cells from all cultivation modes.

The measured cell diameters varied between 2.5 and 5.9 µm. The only significant difference was observed between the WT and mutant M11 cells during mixotrophic cultivation where the M11 cells were significantly larger (ca. 5.9 µm) than the WT cells (ca. 3.1 µm) (Figure S2, Supplemental).

### 3.5. Analysis of Pigment Composition

**Figure 6:**
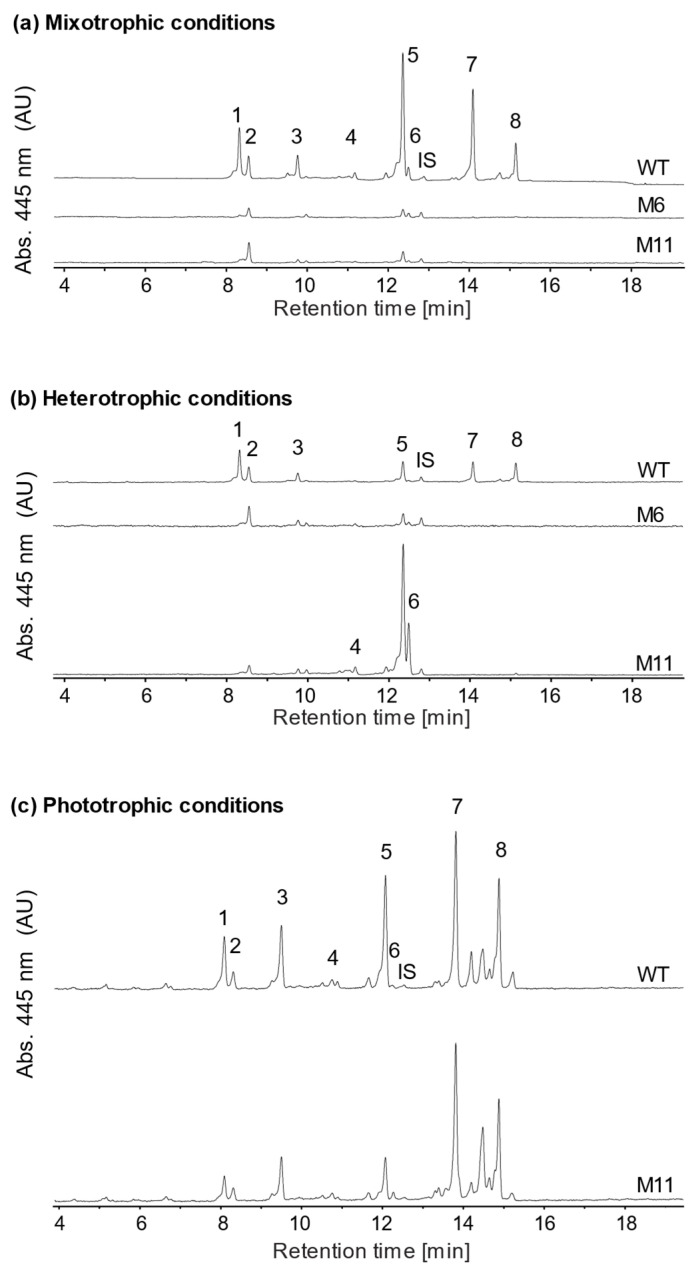
HPLC chromatograms of pigment extracts of *C. vulgaris* wild type (WT), mutants M6 and M11 cultivated both mixotrophically (**a**) and heterotrophically (**b**). M6 did not grow under phototrophic conditions and only data was obtained from WT and M11 (**c**). All samples are normalized to an OD_750_ of 10 hence the relative pigment content can be compared for all strains and cultivations. 1: cis-neoaxanthin, 2: all trans-neoaxanthin, 3: violaxanthin, 4: antheraxanthin, 5: lutein, 6: zeaxanthin, 7: chlorophyll b, 8: chlorophyll a, IS: internal standard (8-apocarotenal).

To investigate how the yellow mutants M6 and M11 were affected in their overall pigment composition, pigments from the two mutants and the WT from both photo-, mixo- and heterotrophic cultivations were extracted and analyzed using HPLC (Figure 6). In Figure S3 the relative quantitative data of the chlorophyll content in all the conditions are shown. The chromatograms revealed that mutant M6 had a severely reduced pigment content relative to the WT at both mixotrophic and heterotrophic cultivations without any detectable chlorophyll a or b (Figure 6). M6 did not grow during phototrophic conditions and therefore the pigments were not analyzed.

In pigment extracts of M11 cultivated in phototrophic conditions both chlorophyll a and b could be detected, and the level of lutein was significantly lower than for the WT (Figure 6 and Figure S3, Supplemental). Zeaxanthin levels were significantly higher in M11 when compared to WT (Figure S3, Supplemental). After seven days of heterotrophic cultivation M11 had significantly higher content of the three carotenoids: zeaxanthin, lutein and antheraxanthin: 20-, 5- and 2-fold higher compared to levels in WT pigment extracts (Figure S3, Supplemental).

### 3.6. Amino Acid Content and Composition

**Figure 7:**
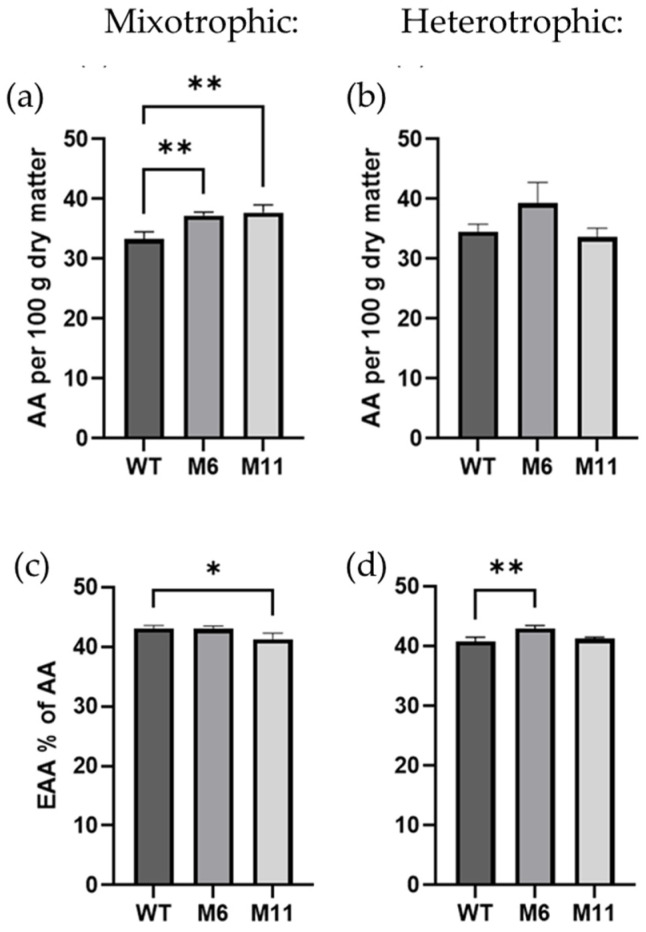
The total content of amino acids and sum of essential amino acids (excluding tryptophane and cysteine) measured for *C. vulgaris* wild type (WT), and two chlorophyll deficient mutants M6 and M11. (**a**) Total content of amino acids (AAs), and (**c**) sum of essential amino acids (EAAs) in % of AA at mixotrophic cultivation. The same parameters measured at heterotrophic cultivation: (**b**) sum of AA and (**d**) content of EAA. All measurements are conducted for biological replicates (n = 3). Significant differences are indicated by *: (*p* > 0.05) and **: (*p* > 0.01).

To investigate potential changes in amino acid content and composition, the amino acid profile was analyzed in WT *C. vulgaris* as well as the M6 and M11 mutants grown mixotrophically and heterotrophically. The total amino acid content (Figure 7a) was significantly higher in both mutants during mixotrophic cultivation, with the WT containing 33.3% AAs (dry weight basis) compared to 37.2% in M6 and 37.7% in M11. There was no significant difference between amino acid content in the three strains when cultivated heterotrophically (Figure 7b). When comparing the fraction of essential amino acids (EAAs) of the total amino acid content, mutant M11 contained slightly less EAA (41.3%) than the wildtype (43.1%) at mixotrophic cultivation conditions (Figure 7c). During heterotrophic cultivation, mutant M6 contained significantly more EAA than the WT (5.2% more) (Figure 7d). An overview of the content of each essential amino acid (mg/g dry matter) for the three *C. vulgaris* strains at mixotrophic and heterotrophic cultivation conditions can be found in the Supplementary Material (Table S1).

### 3.7. Analysis of Cellular Structures Using Cryo-Focused Ion Beam Scanning Microscopy (Cryo FIB-SEM)

**Figure 8:**
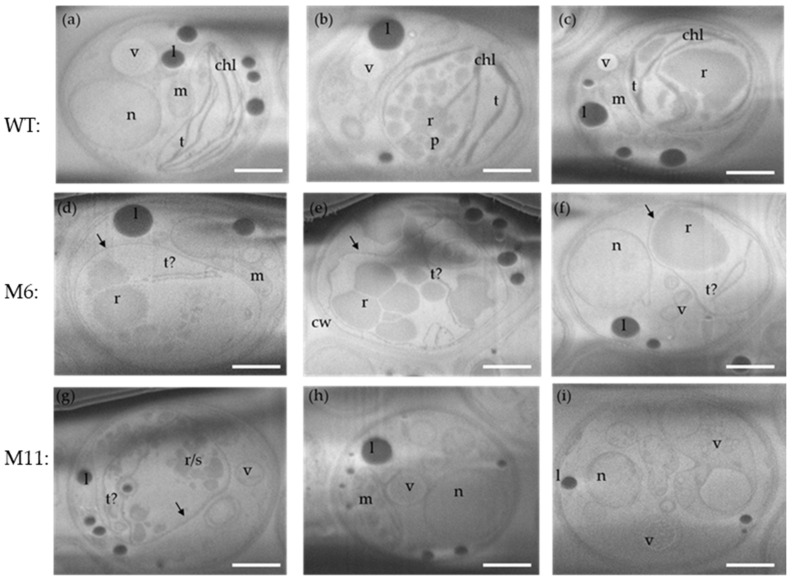
Cryo FIB-SEM micrographs of *C. vulgaris* strains. Three individual cells for each strain, all from heterotrophic cultivations. (**a**–**c**): *C. vulgaris* WT. (**d**–**f**): Mutant M6: chloroplast without thylakoid membranes. Chloroplast membrane (arrow) with fragments of rudimentary thylakoid membranes. No real pyrenoids, only starch and Rubisco-rich matrix. (**g**–**i**): Mutant M11: no chloroplast, only rudimentary membranes indicated by t?. No distinctive pyrenoid, multiple vesicles. Presumptive organelles: chl: chloroplast, t: thylakoid membrane, t?: rudimentary thylakoid membrane, m: mitochondrion, n: nucleus, p: pyrenoid, l: lipid droplet, s: starch, r: Rubisco-rich matrix, v: vesicle, cw: cell wall. Scale bars represent 1 µm. Original micrographs: Figure S5a–i, Supplemental.

Cryo FIB-SEM micrographs were obtained for the three algae strains cultivated under heterotrophic conditions. Ion-beam sequential serial sectioning milling provided visualization of the ultrastructure throughout the entire cell volume generating 3D volume z-stacks of multiple cells of each algal strain. The morphological diversity within each strain is shown in micrographs from three individual cells of both the WT, M6, and M11 (Figure 8).

*C. vulgaris* WT cells all have fully developed chloroplasts with thylakoid membranes and a starch-rich pyrenoid (Figure 8a–c). M6 has a rudimentary chloroplast/plastid encased by an outer membrane but only containing fragments of what appears to be poorly developed thylakoid membranes (Figure 8d–f). Ultrastructures resembling pyrenoids could not be observed, while structures resembling starch grains could be seen.

The ultrastructure of M11 were similar to those of M6 with a few structures resembling rudimentary thylakoid membranes and no distinctive pyrenoid present (Figure 8g–i). Clusters of what is likely to be starch and Rubisco-rich matrix could be observed.

**Figure 9:**
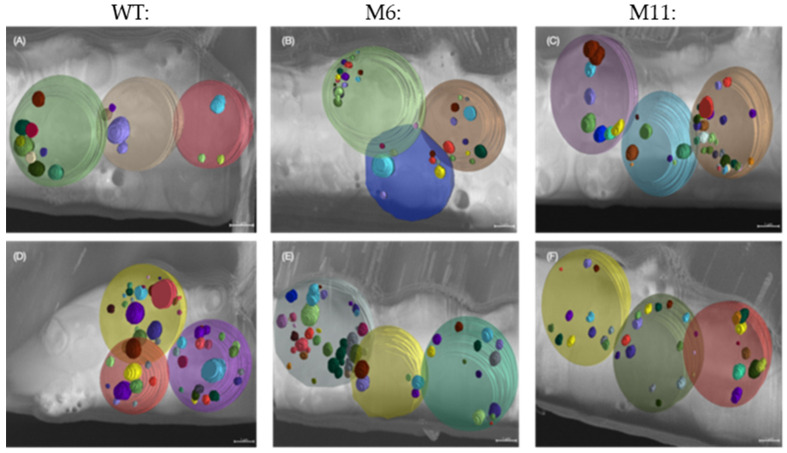
Cryo-Volume Electron Microscopy (cvEM) with 3D visualization of lipids droplets contained in individual cells (**A**) WT cvEM volume 1, (**B**) M6 cvEM volume 1 (**C**) M11 cvEM volume 1 (**D**) WT cvEM volume 2, (**E**) M6 cvEM volume 2 (**F**) M11 cvEM volume 2. Scale bars represent 1 µm.

**Figure 10:**
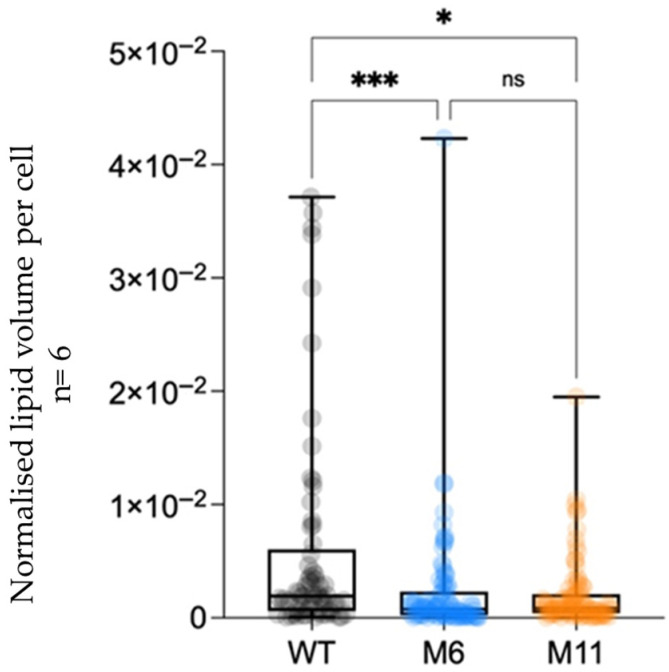
Quantification of total lipid droplet volume normalized to cell volume in *C. vulgaris* wild type (WT) and chlorophyll-deficient mutants M6 and M11, based on cryo FIB-SEM 3D reconstructions. Each point represents the lipid volume fraction of an individual cell (n = 6 cells per strain). Boxes show median and interquartile range, and whiskers indicate the full data range. Statistical differences between strains were assessed using one-way ANOVA. Significance levels: *p* < 0.05 (*), *p* < 0.001 (***), and “ns”: no significant difference.

The 3D volume analysis of the cryo FIB-SEM micrographs illustrate the WT lipid droplets inside three cells of the WT and mutants (Figure 9) and quantification of the lipid droplet to cell volume (Figure 10). See (Figure S4, Supplemental) for an overview of the complete volume with multiple cells and lipid droplets, illustrating the approach used for lipid identification and quantification across whole volumes. The lipid content was also measured in the dry biomass produced from mixotrophic and heterotrophic cultivations (Figure S6, Supplemental). The volume-based observations are consistent with the chemical lipid measurements, showing low and comparable total lipid levels across WT, M6, and M11.

## 4. Discussion

*Chlorella vulgaris* is a highly nutritious green algae and one of the bestselling food-approved microalgae on the market [11]. However, its use as a main food ingredient is impeded by the high chlorophyll content, which contributes to unfavorable organoleptic traits such as a bitter taste and an intense green color disliked by consumers [26,50]. In this study, 12 chlorophyll-deficient mutants of *C. vulgaris* were generated using UVC radiation followed by visual screening. Based on two independent radiation trials, an exposure time of 12–15 s proved optimal for obtaining distinct colonies under the crosslinker settings applied. Based on chlorophyll deficiency, growth performance, and mutation stability, two mutants, M6 and M11, were selected for deeper characterization and compared to WT with respect to biomass productivity, pigment profile, amino acid content, lipid content, and ultrastructure under different trophic modes.

### 4.1. Ultrastructure of the C. vulgaris Cells

Using the recently developed method, Cryo FIB-SEM analysis, we enabled detailed visualization of ultrastructural differences between the strains, all cultivated under heterotrophic conditions. These morphological differences help clarify the physiological impact of the mutations and may guide future identification of the underlying genetic changes. The cryo FIB-SEM data indicate that the Rubisco-rich matrix of the pyrenoid was present in both M6 and M11, despite their poorly developed thylakoid membranes (Figure 8d–i) [51]. This may explain the largely unchanged amino acid content, as protein levels remained comparable across strains. The limited thylakoid-like structures observed in the mutants were often oriented toward the rudimentary pyrenoid, suggesting that thylakoid–pyrenoid connections form early during chloroplast development in *C. vulgaris*, consistent with their functional role in linking the light-dependent and light-independent reactions [51,52].

Cell size varied across trophic modes, with M11 cells significantly larger than WT and M6 during mixotrophic growth (Figure 5 and Figure 6; Figure S1, Supplemental). Cryo-volume EM further revealed differences in lipid droplet distribution between strains. WT cells contained fewer but larger lipid bodies, while mutants contained many smaller droplets, consistent with the biochemical lipid analysis. The use of vitrified samples and volume-based EM analysis minimized artifacts associated with traditional EM preparation, allowing more reliable quantitative comparisons.

### 4.2. Pigment Content and Composition

Pigment analysis showed that both mutants remained chlorophyll-deficient under mixotrophic and heterotrophic conditions. During heterotrophic growth, M11 accumulated markedly higher levels of the carotenoid zeaxanthin, lutein, and antheraxanthin compared to M6 and WT, with zeaxanthin increasing up to 25-fold. Such high carotenoid accumulation is typically associated with high light intensities [53]. Both zeaxanthin and antheraxanthin are formed through the xanthophyll cycle, driven by the enzymes violaxanthin de-epoxidase (VDE) and zeaxanthin epoxidase (ZE) [54,55]. Although xanthophyll accumulation is generally light-induced, carotenoid accumulation in darkness has been reported in other studies [56,57], often associated with oxygen limitation or metabolic constraints. In this study, such stresses are unlikely because the medium used is highly nutrient-rich and contains no NaCl [39].

Mutant M6 lost the ability to synthesize chlorophyll and did not grow phototrophically. M11 exhibited strongly reduced pigment levels in light and remained chlorophyll-deficient in mixotrophic and heterotrophic conditions. This phenotype may reflect a mutation in one of the chlorophyll biosynthetic genes. Alternatively, catabolite repression may play a role, as glucose can suppress specific steps in tetrapyrrole biosynthesis in some algae [58,59]. Further studies are needed to clarify the mechanism behind M11’s phenotype.

The strong carotenoid accumulation in M11 under heterotrophic conditions is noteworthy. Because carotenoids can reduce photosynthetic efficiency, their accumulation is usually physiologically costly in phototrophic systems [57]. Heterotrophic cultivation of mutants like M11 therefore offers a promising non-GMO strategy for industrial pigment production.

### 4.3. Biomass Productivity

Phototrophic growth of WT was low but comparable to previous studies using similar light intensities [60]. As expected, M6 did not grow phototrophically, while M11 produced some chlorophyll and biomass in light, although substantially less than WT. M11 produced almost 50% less biomass under mixotrophic than heterotrophic conditions (3.1 vs. 5.8 g dm/L), potentially due to ROS formation caused by accumulating chlorophyll intermediates [61]. Biomass productivities obtained in this study are consistent with earlier reports on pigment-deficient *C. vulgaris* mutants [24,33,34]. Typically, WT strains grow best under mixotrophic conditions due to combined use of organic carbon and light [15]. However, the use of heterotrophically adapted precultures may have influenced growth performance across trophic modes. Furthermore, application of mixotrophic conditions does not guarantee simultaneous use of organic carbon and light [13].

### 4.4. Biomass Composition

During mixotrophic cultivation, total amino acid content was significantly higher in the mutants (37.2% for M6 and 37.7% for M11) compared to WT (33.7%). Under heterotrophic growth, no significant differences were observed. These values align with those reported in other pigment-deficient *Chlorella* strains [34]. Higher protein contents reported in earlier studies [24] likely reflect the use of a nitrogen-to-protein conversion factor of 6.25, which overestimates true protein content in green microalgae compared to the more accurate factor of ~5.5 [62,63]. Essential amino acid (EAA) proportions were also comparable to earlier studies [33,61], reaching values similar to those in eggs and meat [64]. Lipid contents were low (3.1–4.1%), consistent with growth in nutrient-rich medium and with cryo FIB-SEM observations showing differing lipid droplet distributions between mutants and WT (Figure 9 and Figure 10, and Figure S6 Supplemental). The disrupted thylakoids in M6 and M11 may have affected lipid biosynthesis pathways, leading to reduced lipid content and altered droplet morphology. The remaining biomass mass balance suggests that all strains contained high levels of carbohydrates, consistent with observations of abundant starch granules in the cryo FIB-SEM micrographs.

### 4.5. Limitations, Robustness, and Scalability

Ultrastructural analyses in this study were performed on heterotrophically grown cells, as this trophic mode aligns directly with the intended application of the developed strains. While future work should compare ultrastructure across phototrophic and mixotrophic conditions to fully resolve chloroplast and thylakoid development under different trophic modes, such analyses lie beyond the applied focus of the present study.

All precultures were adapted to heterotrophic growth prior to experimentation. This was a deliberate and biologically justified choice since the aim of the work was to breed chlorophyll-deficient *C. vulgaris strains* optimized for heterotrophic cultivation, where chlorophyll and functional thylakoids are neither required nor desired. For this purpose, maintaining the cells in a heterotrophic metabolic state throughout the workflow was both natural and appropriate.

Although the mutants did not outperform WT under phototrophic growth and M6 was unable to grow in light, this is fully consistent with the objectives of the study. The intended industrial application is heterotrophic cultivation for food ingredient production, where light-independent growth, reduced pigmentation, and high productivity are the relevant performance criteria. Validation of strain robustness at production scale including oxygen transfer, contamination control, and high-density fermentation represents an important next step beyond the scope of the present work.

## 5. Conclusions

This study generated chlorophyll-deficient mutants of *C. vulgaris* using UVC mutagenesis and identified two stable strains (M6 and M11) for detailed characterization. Both mutants remained pigment-deficient under mixotrophic and heterotrophic growth; only M11 produced limited chlorophyll when grown in phototropic conditions. Despite disrupted thylakoid structure, both mutants maintained biomass productivity and amino acid profiles comparable to the wild type under heterotrophic cultivation. M11 accumulated high carotenoid levels during heterotrophic growth, highlighting potential for pigment production in darkness. Overall, simple non-GMO mutagenesis combined with visual screening can yield *C. vulgaris* strains with reduced color intensity and preserved nutritional value, supporting improved consumer acceptance of microalgae-based food ingredients.

## Figures and Tables

**Table 1:** Overview of the visual phenotypes of the 12 mutants selected from the two UVC-radiation trials.

Mutant	Mutagenesis	Colour, Heterotrophic
**M1**	1st trial	Orange/red
**M2**	1st trial	Lime green
**M3**	1st trial	Lime green
**M4**	1st trial	Yellow
**M5**	1st trial	Orange/red
**M6**	1st trial	Light orange
**M7**	1st trial	Almost white, very poor growth
**M8**	1st trial	Yellow
**M9**	2nd trial	Light green
**M10**	2nd trial	Orange, reverted to green
**M11**	2nd trial	Orange
**M12**	2nd trial	Orange

## Data Availability

The raw data supporting the conclusions of this article will be made available by the authors on request.

## Supplementary Materials

The following supporting information can be downloaded at https://www.mdpi.com/article/10.3390/bioengineering13030318/s1, Figure S1: Original micrographs for Figure 5; Figure S2: The average cell size for each strain: Chlorella vulgaris wild type and the chlorophyll deficient mutants M11 and M6; Figure S3: Bar graphs illustrating the difference in pigment content of the two mutants M6 and M11 relative to the wildtype (constant in both graphs); Figure S4. Representative cryo FIB-SEM 3D volume visualization showing all lipid droplets and cells contained in an entire 125 μm^3^ volume through a wild type Chlorella vitrified sample; Figure S5a: Original Cryo FIB-SEM micrograph for Figure 8a; Figure S5b: Original Cryo FIB-SEM micrograph for Figure 8b; Figure S5c: Original Cryo FIB-SEM micrograph for Figure 8c; Figure S5d: Original Cryo FIB-SEM micrograph for Figure 8d; Figure S5e: Original Cryo FIB-SEM micrograph for Figure 8e; Figure S5f: Original Cryo FIB-SEM micrograph for Figure 8f; Figure S5g: Original Cryo FIB-SEM micrograph for Figure 8g; Figure S5h: Original Cryo FIB-SEM micrograph for Figure 8h; Figure S5i: Original Cryo FIB-SEM micrograph for Figure 8i; Figure S6: Lipid content in C. vulgaris WT and the two mutants M6 and M11 biomass; Table S1: Essential amino acid (EAA) mg/g biomass (dry matter) for C. vulgaris wild type and the two chlorophyll deficient mutants M6 and M11 cultivated mixotrophically (mix) and heterotrophically (het).

## Abbreviations

The following abbreviations are used in this manuscript:

AA	Amino Acids
AMST	Alignment to Median Smoothed Template
ANOVA	Analysis of Variance
CCAP	Culture Collection of Algae and Protozoa
cvEM	Cryo Volume Electron Microscopy
Cryo FIB-SEM	Cryo Focused Ion Beam Scanning Electron Microscopy
EAA	Essential Amino Acids
EMS	Ethyl Methane Sulphonate
ESI	Electrospray Ionization
HPLC	High Performance Liquid Chromatography
LC-MS	Liquid Chromatography–Mass Spectrometry
OD_750_	Optical Density at 750 nm
PVDF	Polyvinylidene Fluoride
ROS	Reactive Oxygen Species
UHPLC	Ultra High Performance Liquid Chromatography
UVC	Ultraviolet C
CVDE	Violaxanthin De-epoxidase
WT	Wild Type
ZE	Zeaxanthin Epoxidase

## Appendix A. Recipe for the Modified P4-TES Medium Applied in This Study

Base medium: prepare 1 L of P4-TES medium according to the original formulation described by Lippi et al. (2018) [39].

Glucose addition: add 18 g glucose (0.1 M) before autoclaving for mixotrophic or heterotrophic cultivation.

Vitamin supplementation: after cooling, add vitamins by sterile filtration:

Vitamin B1 (thiamine): Prepare a stock of 0.12 g thiamine hydrochloride in 100 mL distilled water. Add 1ml stock to achieve a final concentration of 3.6 µM.

Vitamin B12 (cyanocobalamin): Prepare a stock of g cyanocobalamin in 100 mL distilled water. Add 1 mL stock to achieve a final concentration of 0.738 µM.
