# Supplementary material for: Breeding Chlorophyll-Deficient Mutants of Chlorella vulgaris to Enhance Consumer Acceptance
Source: Bioengineering (Basel). 2026 Mar 10;13(3):318. doi: 10.3390/bioengineering13030318 (PMC13024007; doi:10.3390/bioengineering13030318)
Supplement: Supplementary file 1 [file bioengineering-13-00318-s001.zip › bioengineering-4133006-supplementary.pdf]

Supplementary

Breeding Chlorophyll-Deficient Mutants of *Chlorella vulgaris* to Enhance Consumer Acceptance

Malene Lihme Olsen, Daniel Poveda-Huertes, Duygu Ozcelik, Emil Gundersen, Jens Frederik Bang Thøfner, Maryna Kobylynska, Stefania Marcotti, Roland A. Fleck, Damien McGrouther, Johan Andersen-Ranberg, Charlotte Jacobsen & Poul Erik Jensen

January 2026

Table S1. Essential amino acid (EAA) mg/g biomass (dry matter) for *C. vulgaris* wild type and the two chlorophyll deficient mutants M6 and M11 cultivated mixotrophically (mix) and heterotrophically (het).

EAA mg/g biomass (dm)	WT mix	M6 mix	M11 mix	WT het	M6 het	M11 het
Phenylalanine	12.9 ± 0.7	11.9 ± 0.9	10.2 ± 0.4	9.7 ± 0.5	12.2 ± 1.8	9.7 ± 0.6
Leucine	56.6 ± 2.8	61.3 ± 4.0	56.5 ± 3.7	49.3 ± 3.6	62.3 ± 7.9*	47.1 ± 3.7
Isoleucine	14.1 ± 0.6	15.0 ± 0.1	14.7 ± 0.7	13.4 ± 0.6	16.8 ± 1.5*	13.8 ± 0.4
Methionine	3.0 ± 0.5	5.0 ± 0.4	4.1 ± 0.9	4.4 ± 1.0	4.6 ± 1.6	4.8 ± 0.9
Valine	20.2 ± 0.8	22.9 ± 0.2	23.2 ± 1.0	21.2 ± 0.8	25.4 ± 2.1*	21.5 ± 0.7
Threonine	15.2 ± 1.4	17.4 ± 0.9	17.8 ± 1.1	16.7 ± 0.7	18.1 ± 1.4	16.3 ± 0.7
Histidine	6.3 ± 0.2	8.3 ± 0.3*	7.7 ± 0.2*	7.1 ± 0.4	7.9 ± 0.7	7.3 ± 0.4
Lysine	15.2 ± 0.5	20.0 ± 0.3*	21.5 ± 1.4*	19.1 ± 1.2	21.6 ± 2.7	18.2 ± 0.3

Figure S1.

Original micrographs for figure 5.

Cells after 7 days of cultivation at three trophic modes. (a-c) Mutant M11 cultivated photo- (a), mixo- (b) and heterotrophically (c). (d-f) Mutant M6 cultivated photo- (d), mixo- (e) and heterotrophically (f). (g-i) wild type strain cultivated at photo- (g), mixo- (h) and heterotrophic conditions (i).

Light microscopy 1000x magnification.

Scale bar represents 10µm

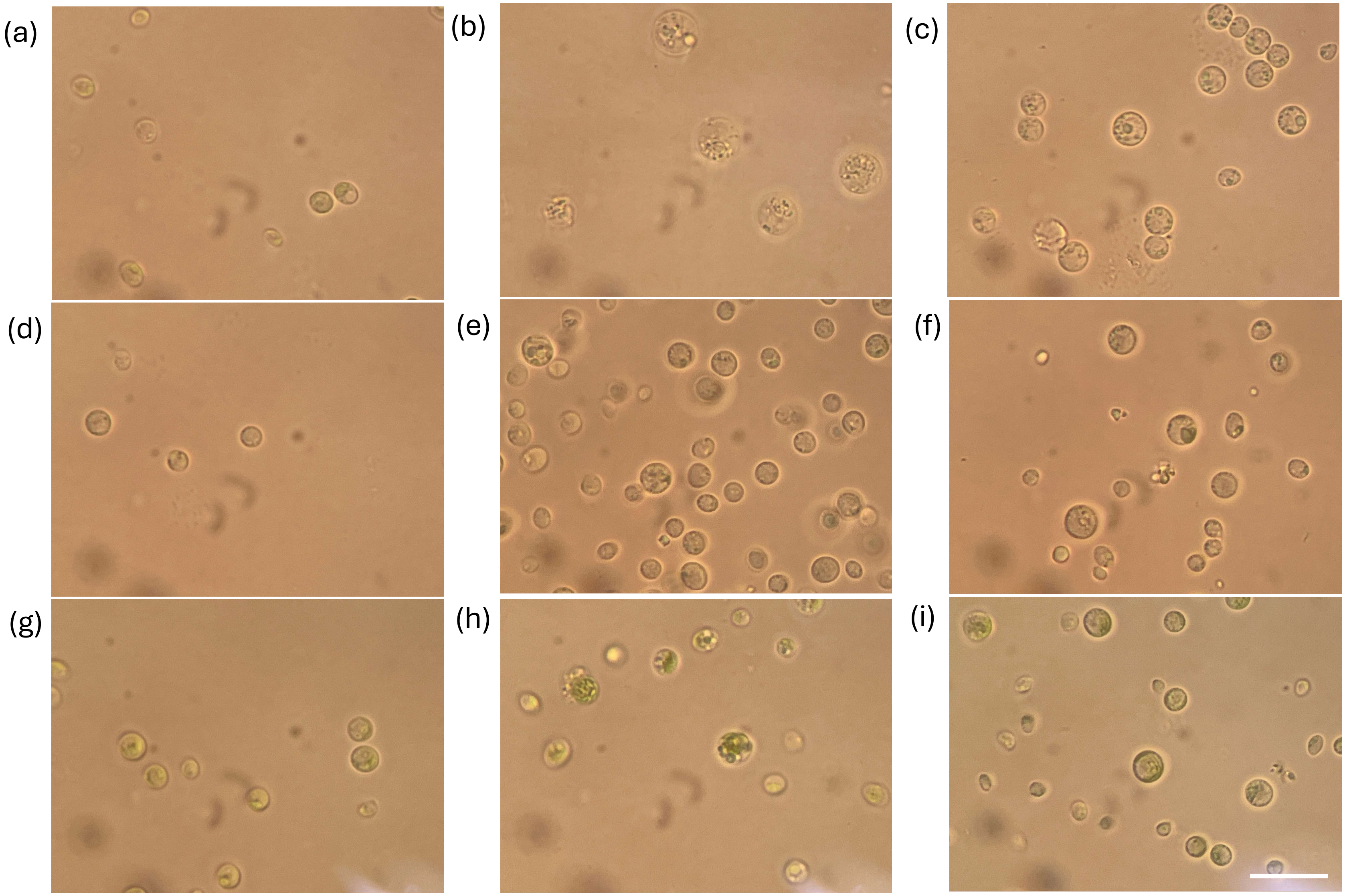

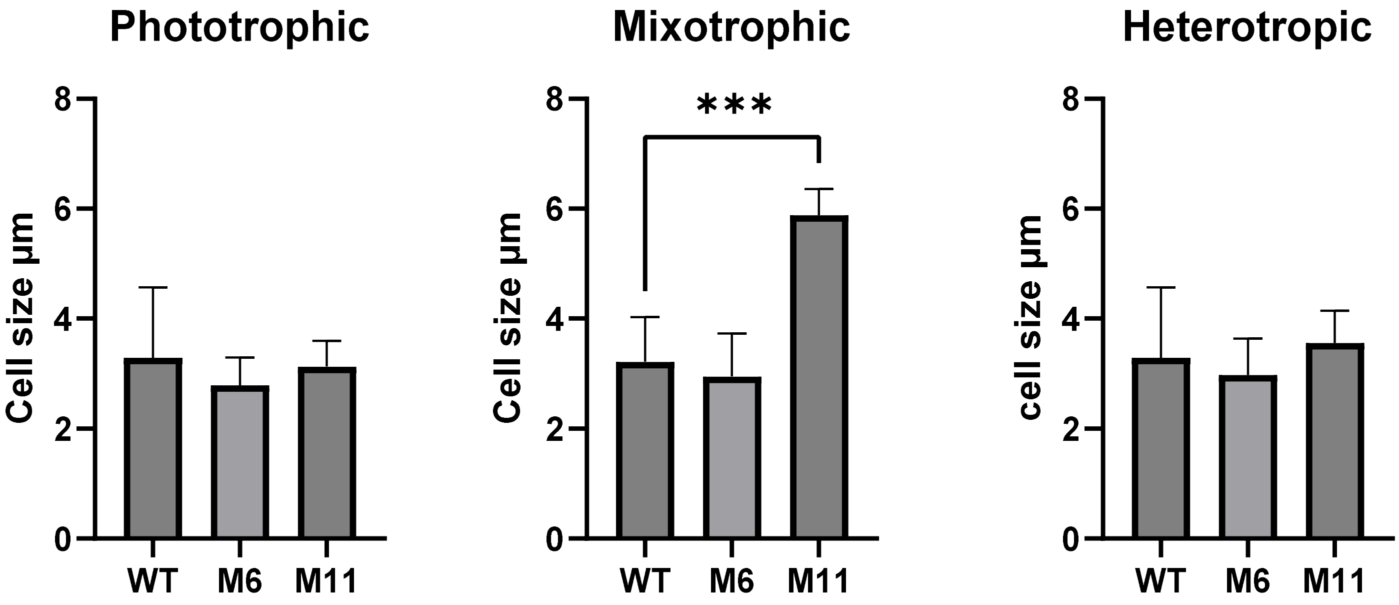

Figure S2. The average cell size for each strain: *Chlorella vulgaris* wild type and the chlorophyll deficient mutants M11 and M6. Sizes are normally distributed, and one-way ANOVA analysis indicates that the average cell size of mutant M11 is significantly larger than the wild type but only at mixotrophic cultivation conditions. n = 4-8 cells. Significant difference indicated as: *** ($p < 0.005$).

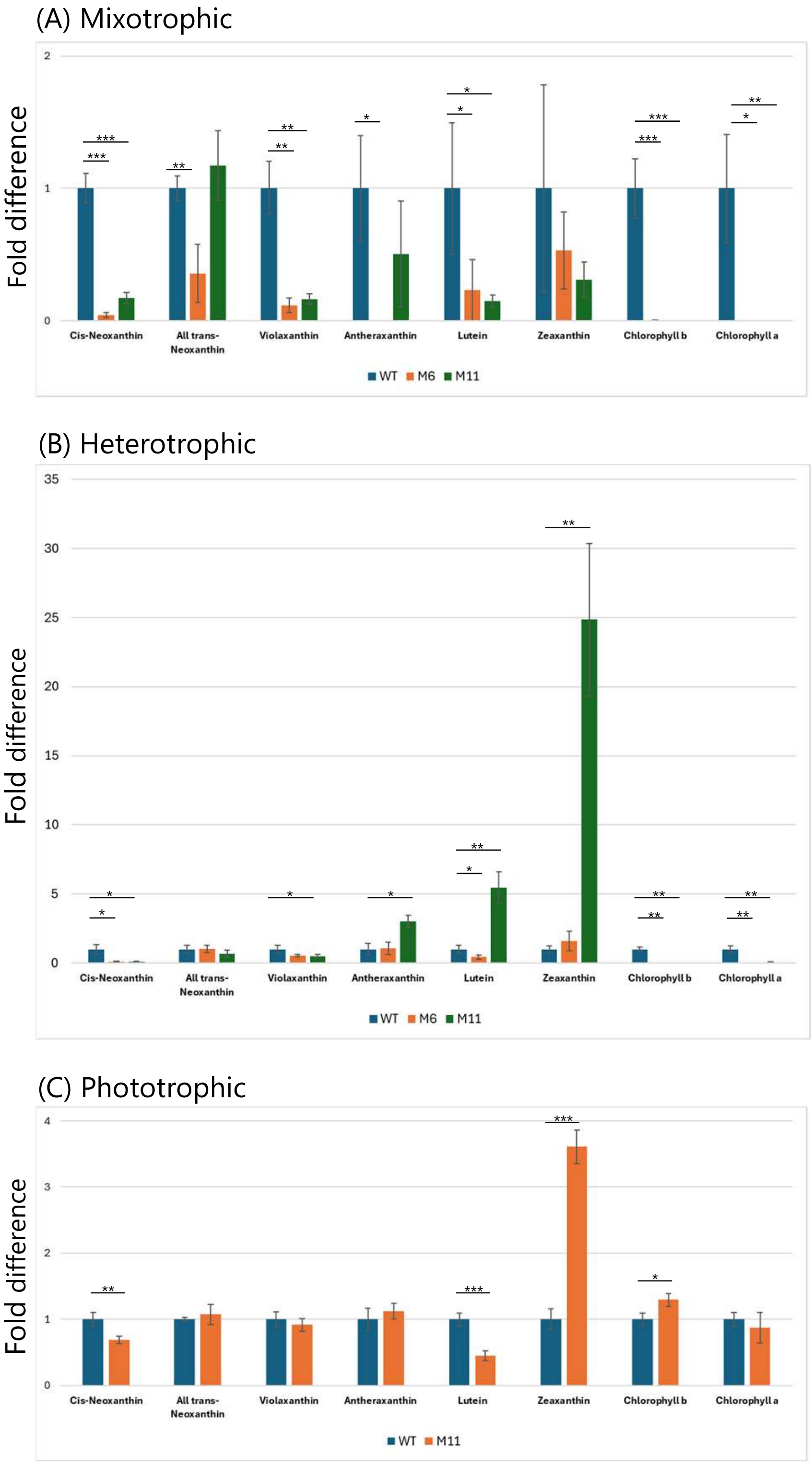

Figure S3. Bar graphs illustrating the difference in pigment content of the two mutants M6 and M11 relative to the wildtype (constant in all graphs). All samples are normalized to $OD_{750} = 10$. Shown is for mixotrophic cultivation (A), heterotrophic cultivation (B) and phototrophic cultivation (C). M6 did not produce any biomass for analysis during phototropic cultivation. Significant differences between wild type and mutants are indicated: * $p < 0.05$, ** $p < 0.01$ and ***$p < 0.001$.

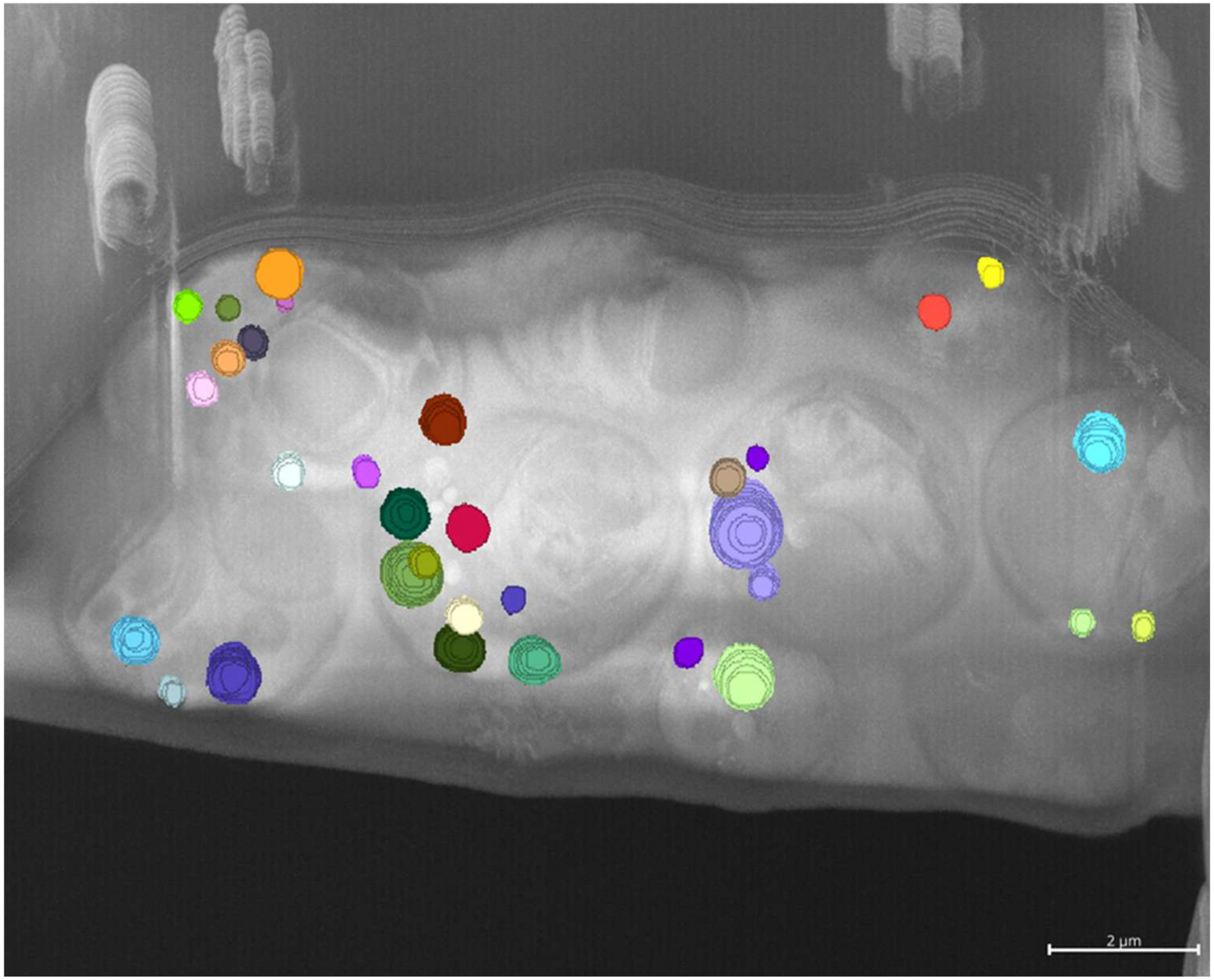

Figure S4. Representative cryo FIB-SEM 3D volume visualization showing all lipid droplets and cells contained in an entire 125 µm3 volume through a wild type *Chlorella* vitrified sample. Scale bar represents 2 µm.

Figure S5a.

Original Cryo FIB-SEM micrograph for figure 8a.

Chlorella vulgaris wild type Heterotrophic cultivation.

Scale bar represents 1 μm.

(a)

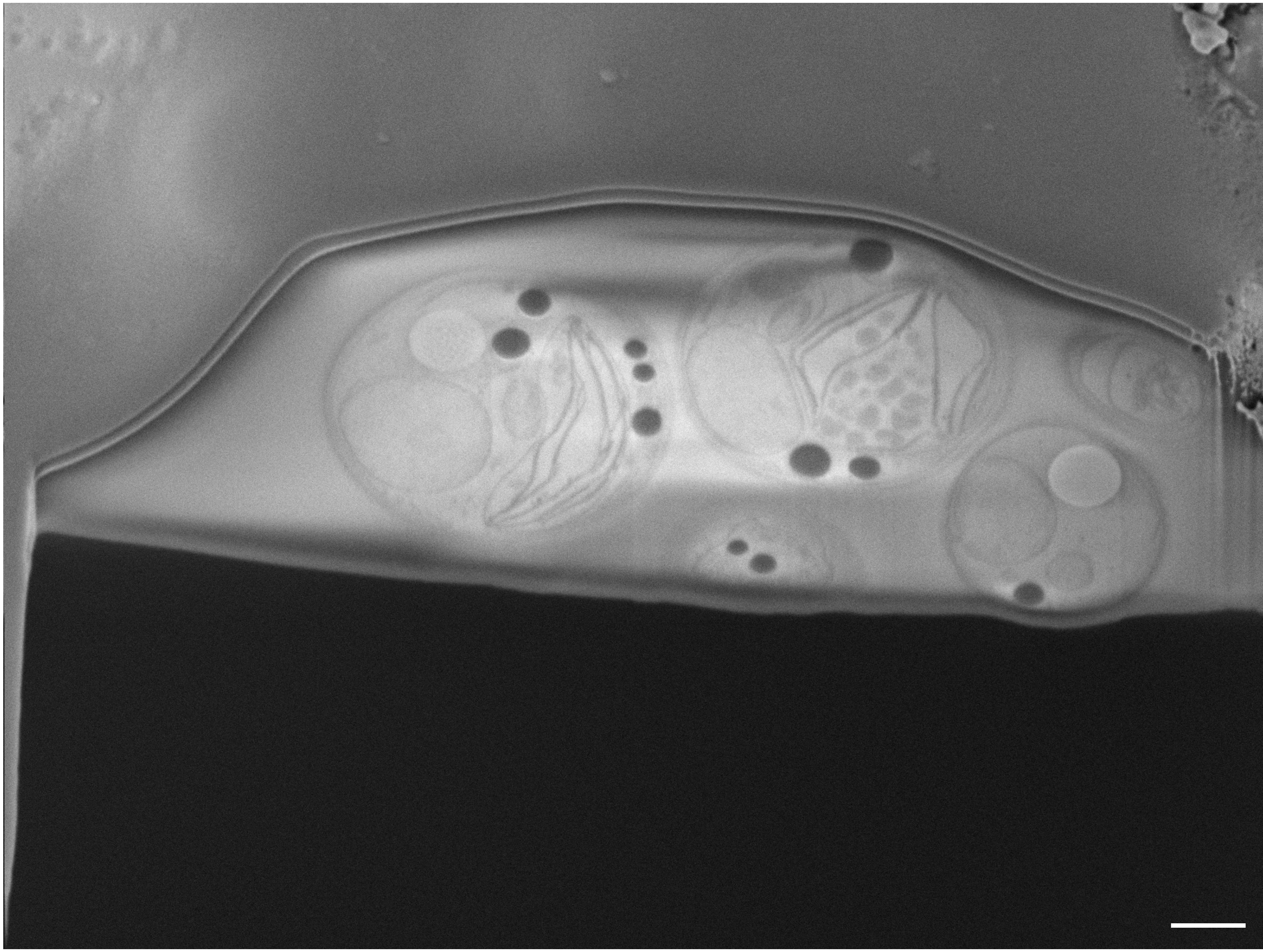

Figure S5b.

Original Cryo FIB-SEM micrograph for figure 8b.

Chlorella vulgaris wild type Heterotrophic cultivation.

Scale bar represents 1 μm.

(b)

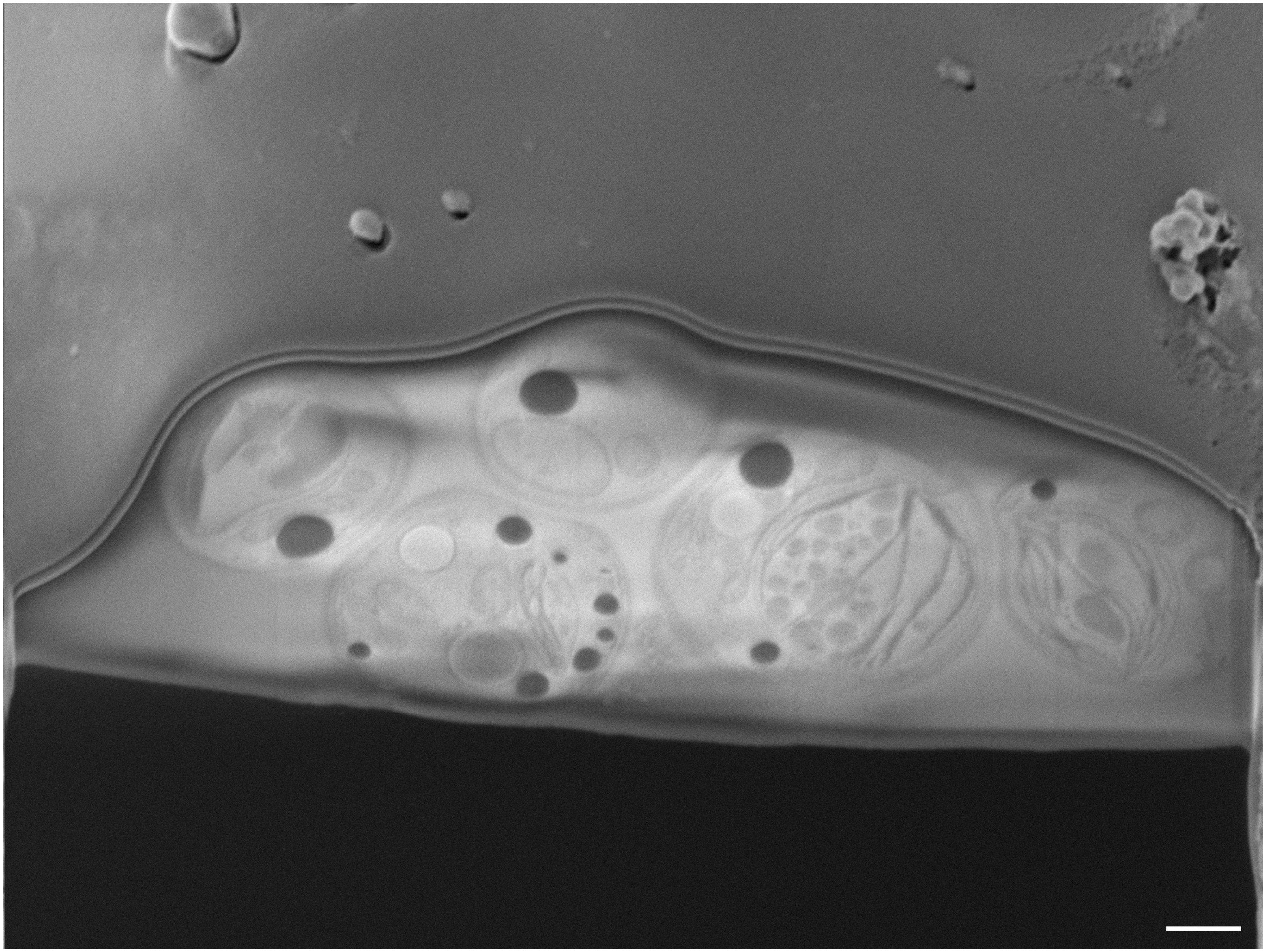

Figure S5c.

Original Cryo FIB-SEM micrograph for figure 8c.

Chlorella vulgaris wild type Heterotrophic cultivation.

Scale bar represents 1 μm.

(c)

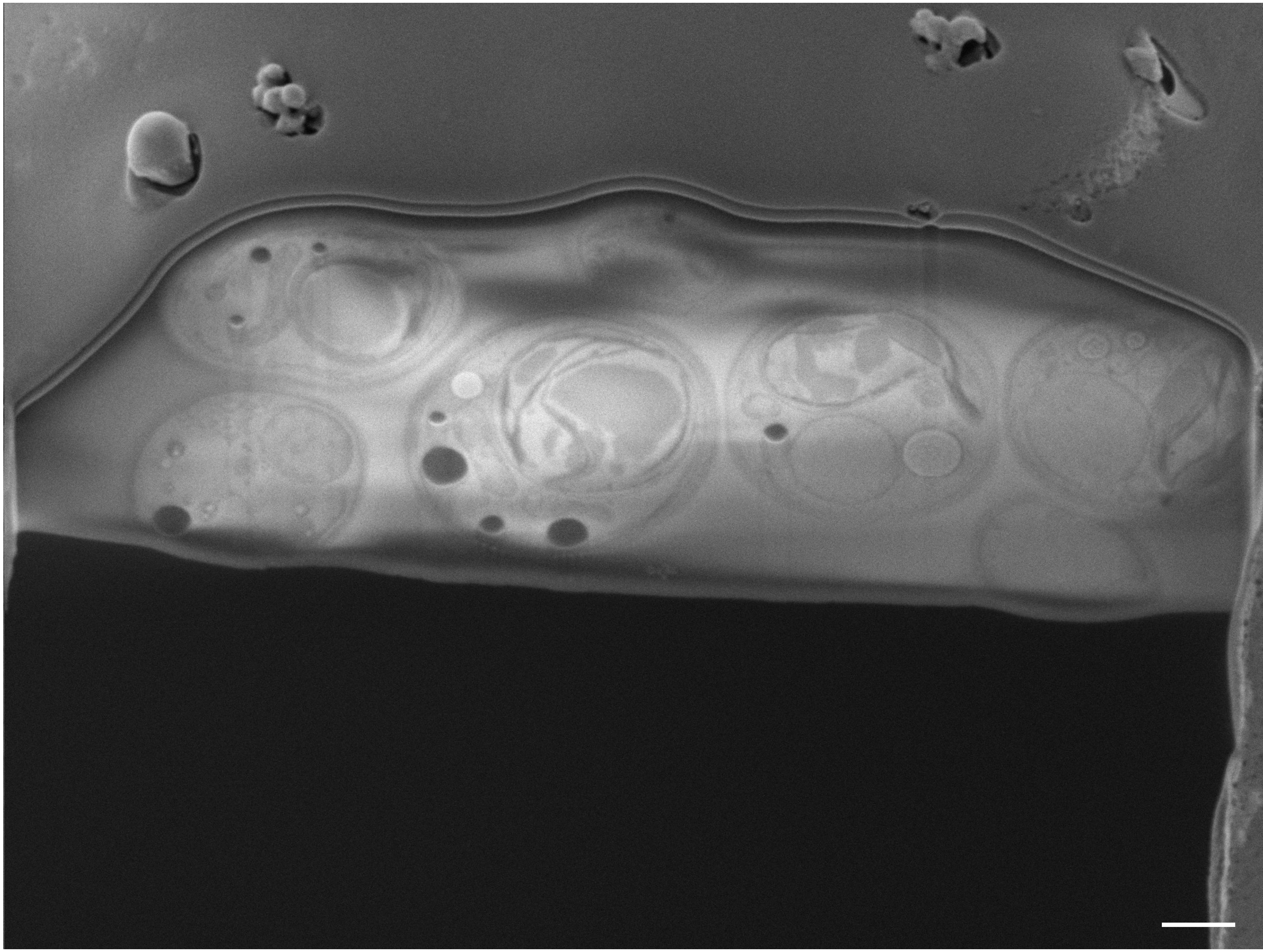

Figure S5d.

Original Cryo FIB-SEM micrograph for figure 8d.

Chlorella vulgaris, mutant M6. Heterotrophic cultivation.

Scale bar represents 1 μm.

(d)

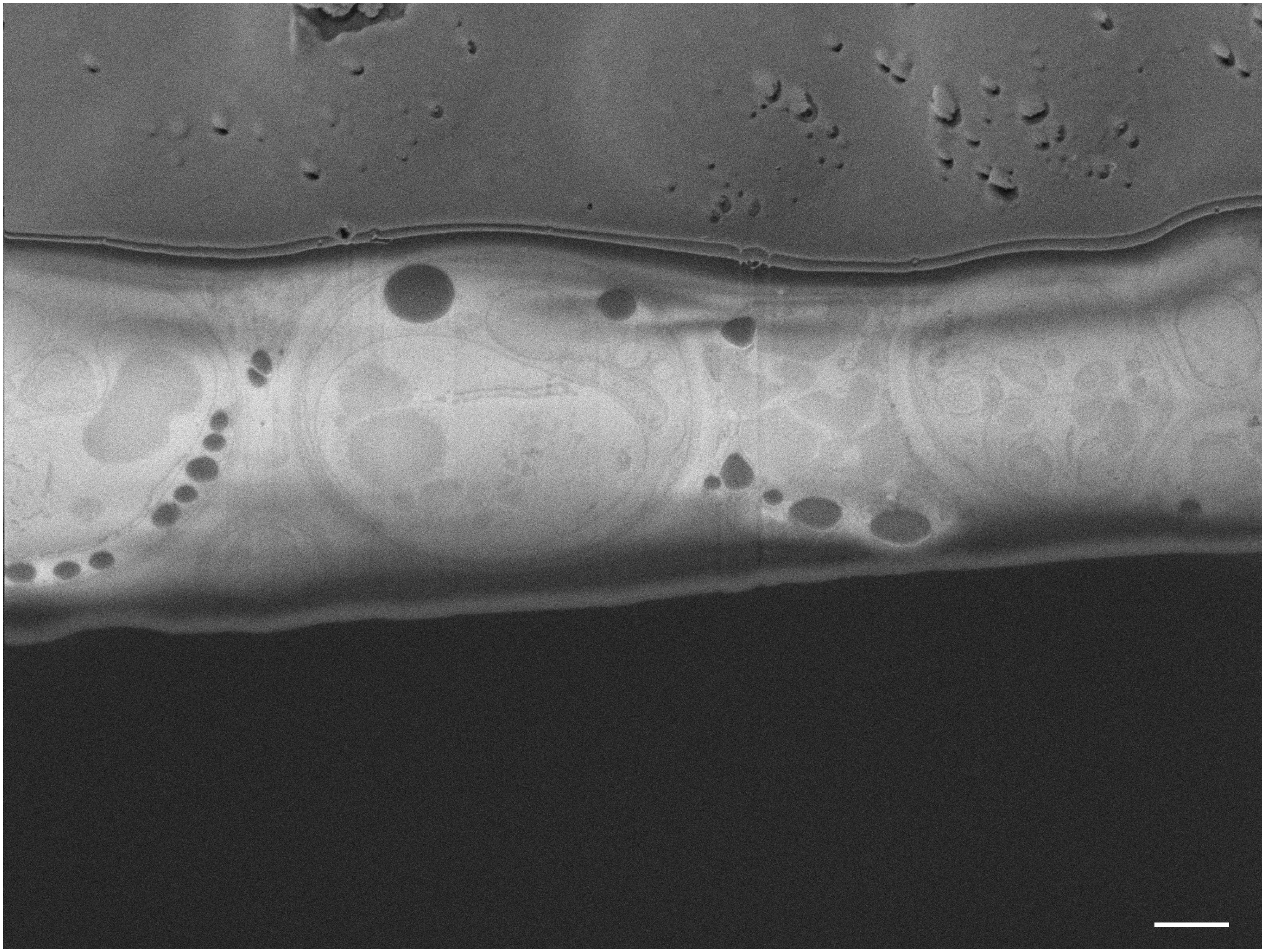

Figure S5e.

Original Cryo FIB-SEM micrograph for figure 8e.

Chlorella vulgaris, mutant M6. Heterotrophic cultivation.

Scale bar represents 1 µm.

(e)

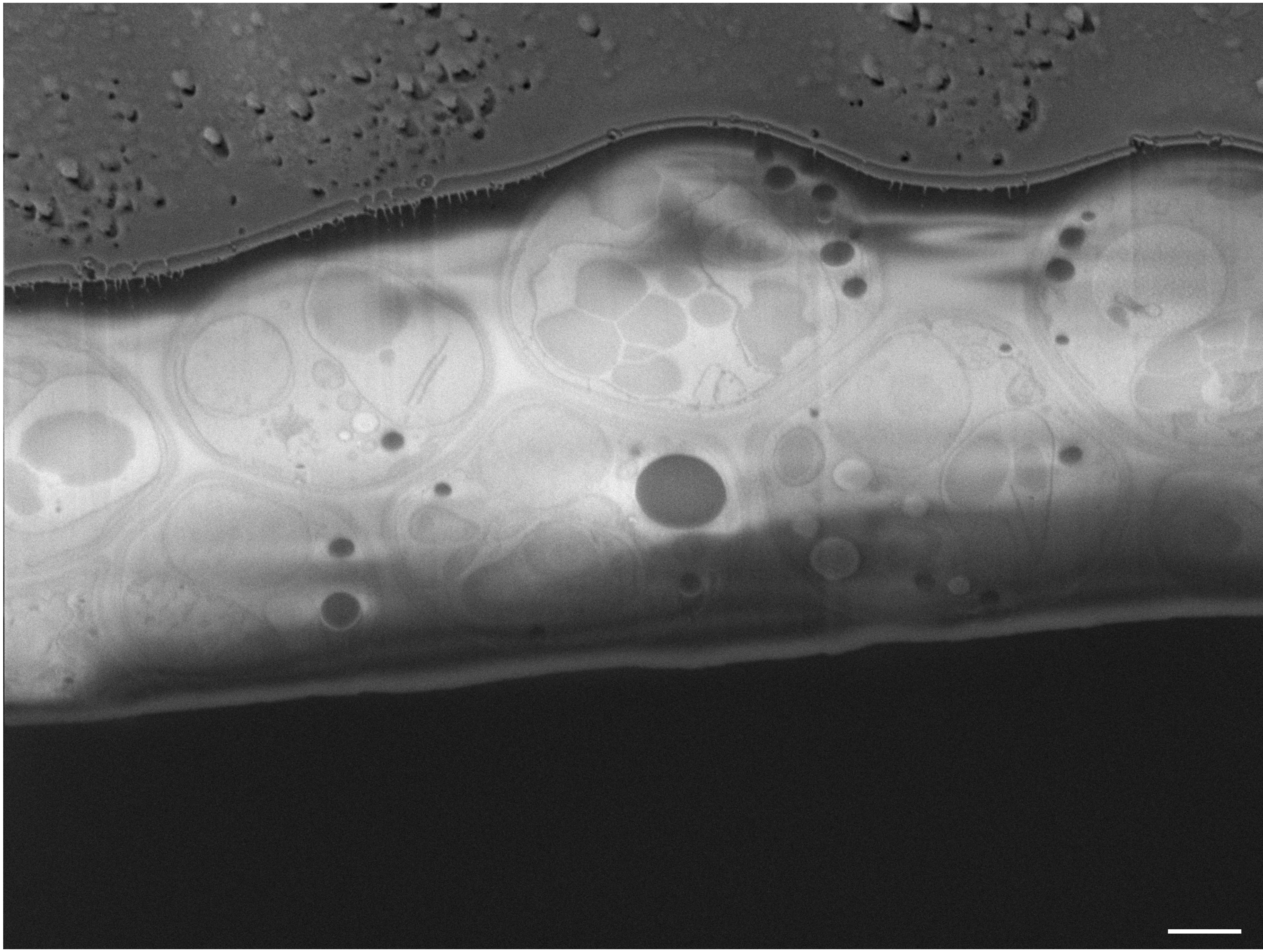

Figure S5f.

Original Cryo FIB-SEM micrograph for figure 8f.

Chlorella vulgaris, mutant M6. Heterotrophic cultivation.

Scale bar represents 1 µm.

(f)

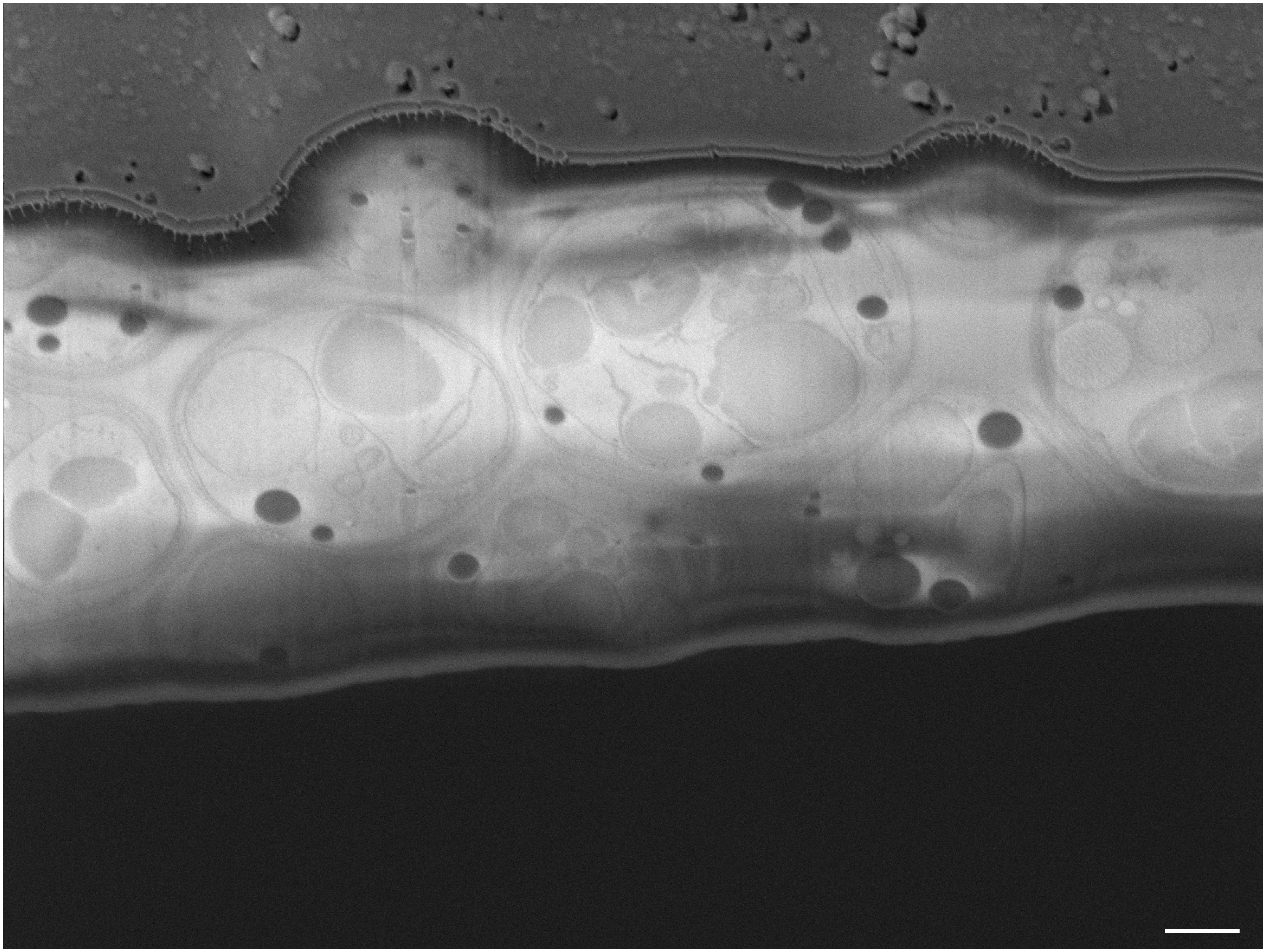

Figure S5g.

Original Cryo FIB-SEM micrograph for figure 8g.

Chlorella vulgaris, mutant M11. Heterotrophic cultivation.

Scale bar represents 1 μm.

(g)

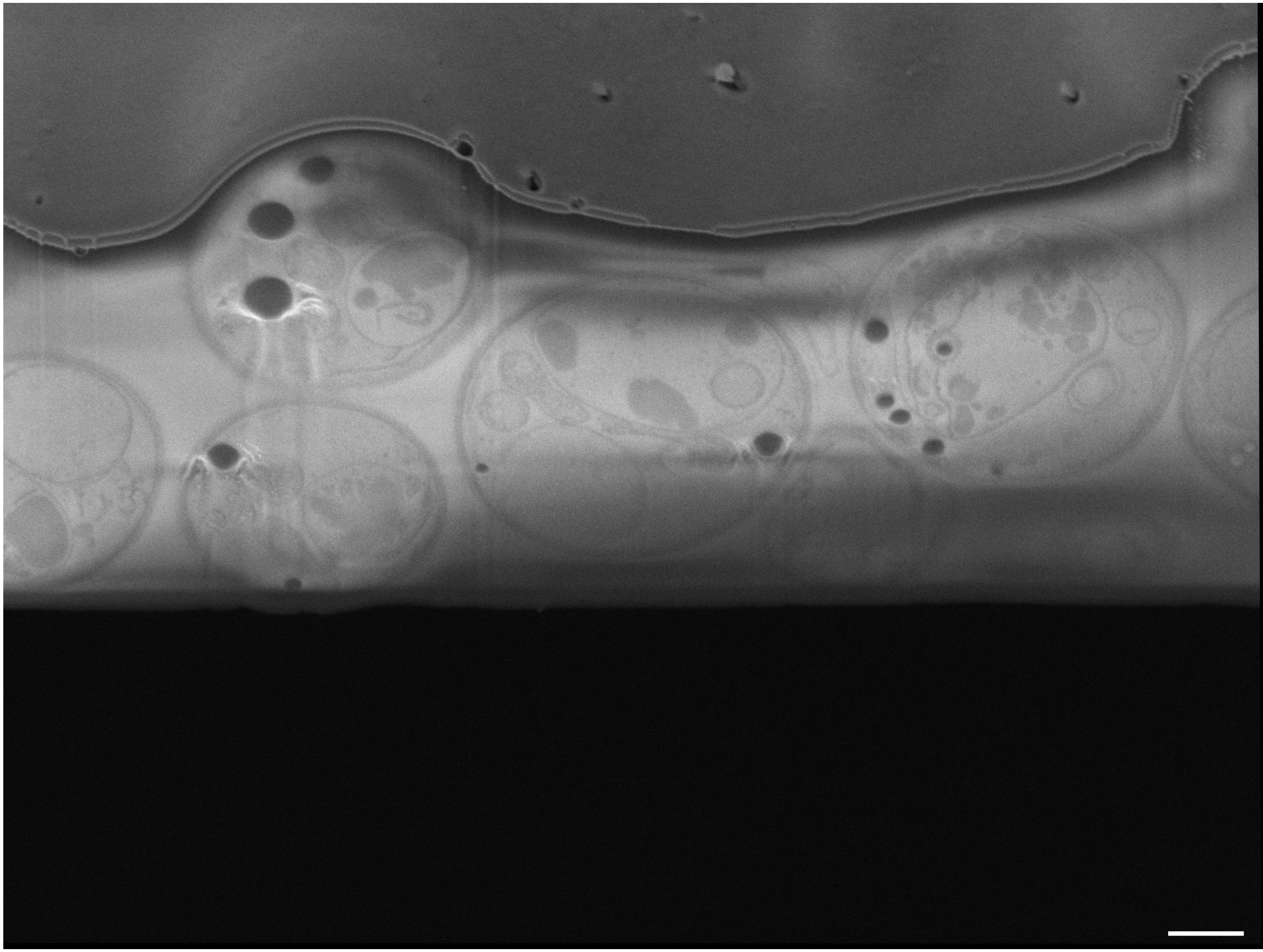

Figure S5h.

Original Cryo FIB-SEM micrograph for figure 8h.

Chlorella vulgaris, mutant M11. Heterotrophic cultivation.

Scale bar represents 1 μm.

(h)

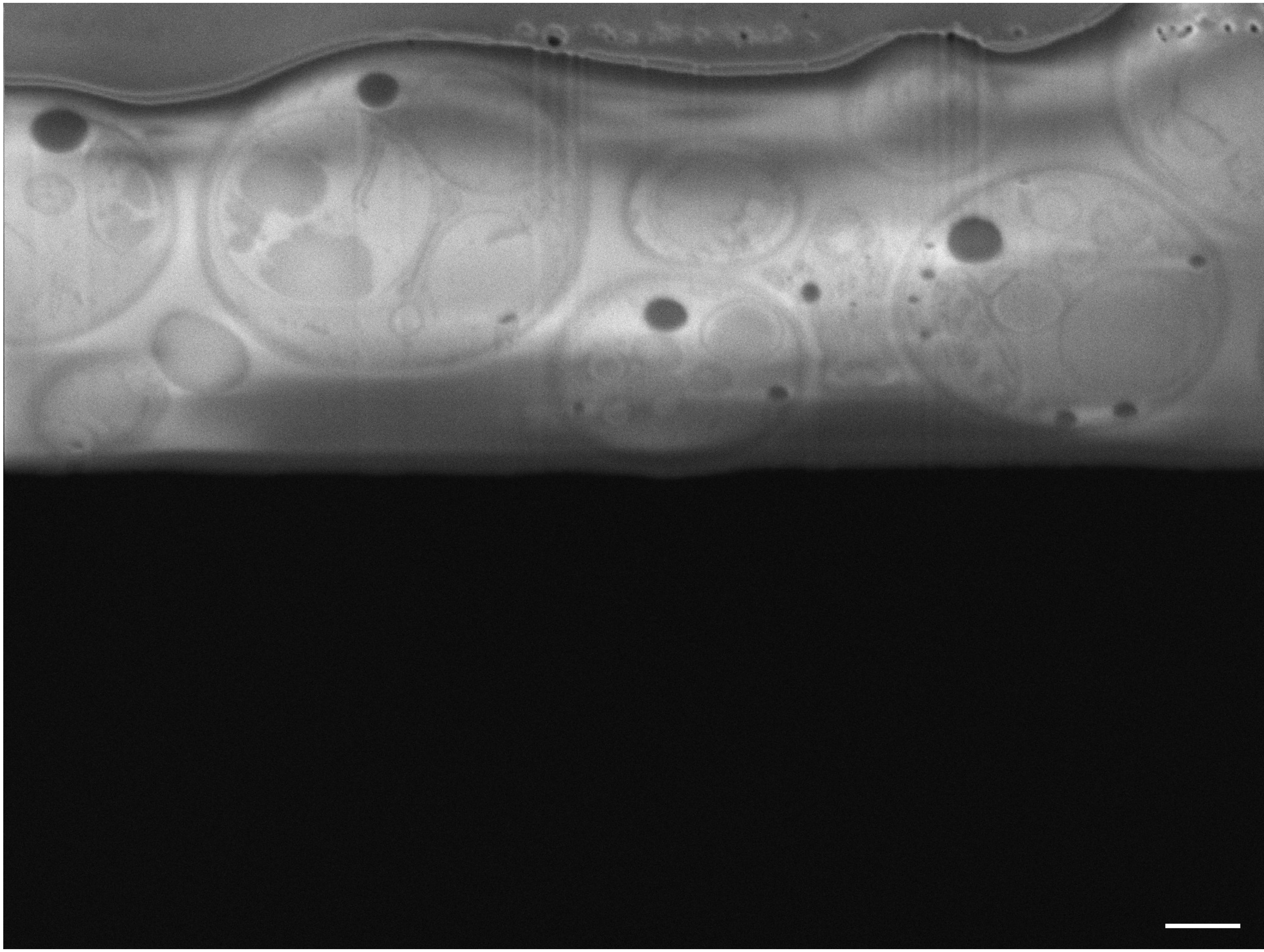

Figure S5i.

Original Cryo FIB-SEM micrograph for figure 8i.

Chlorella vulgaris, mutant M11. Heterotrophic cultivation.

Scale bar represents 1 μm.

(i)

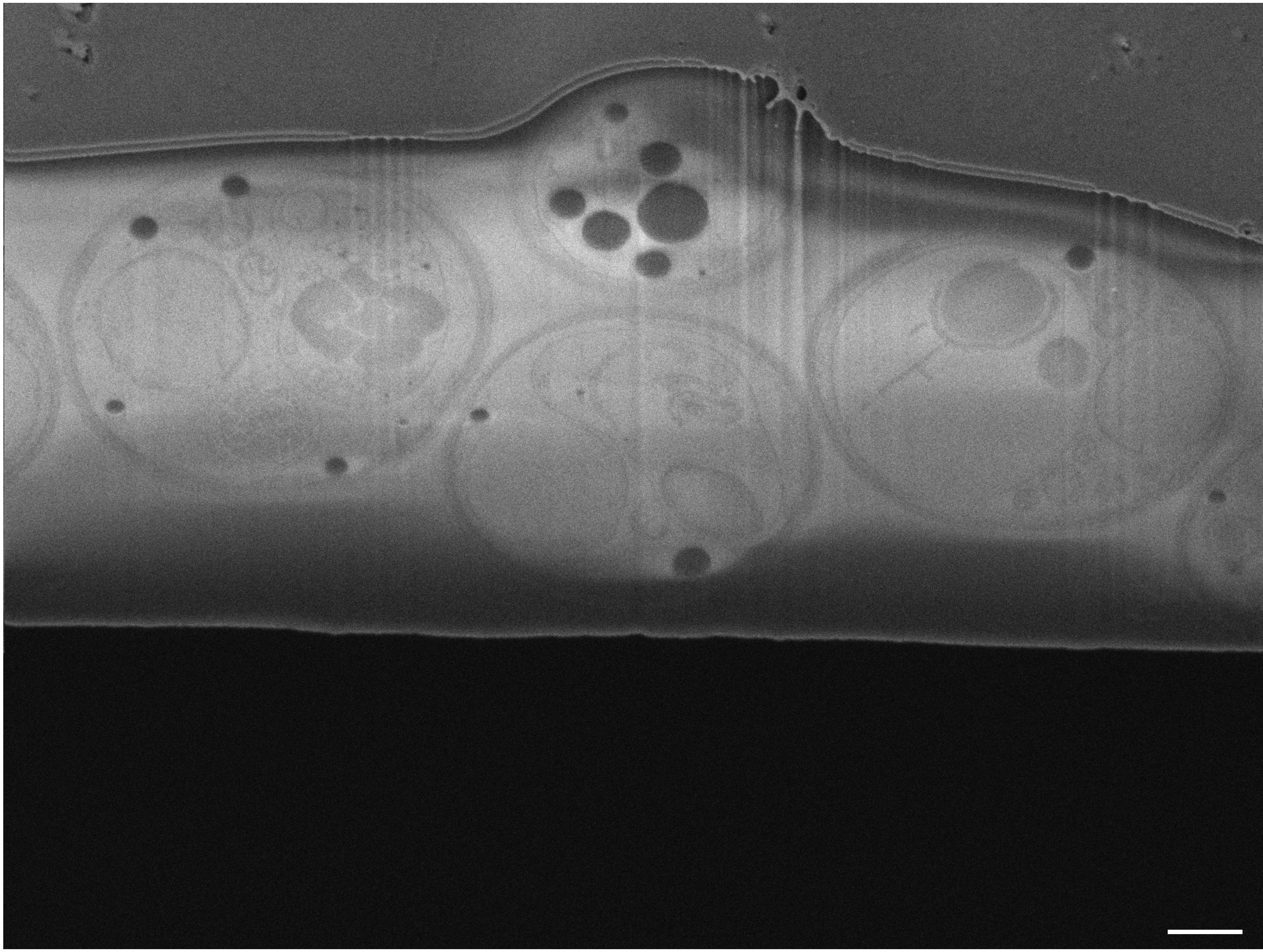

Figure S6.

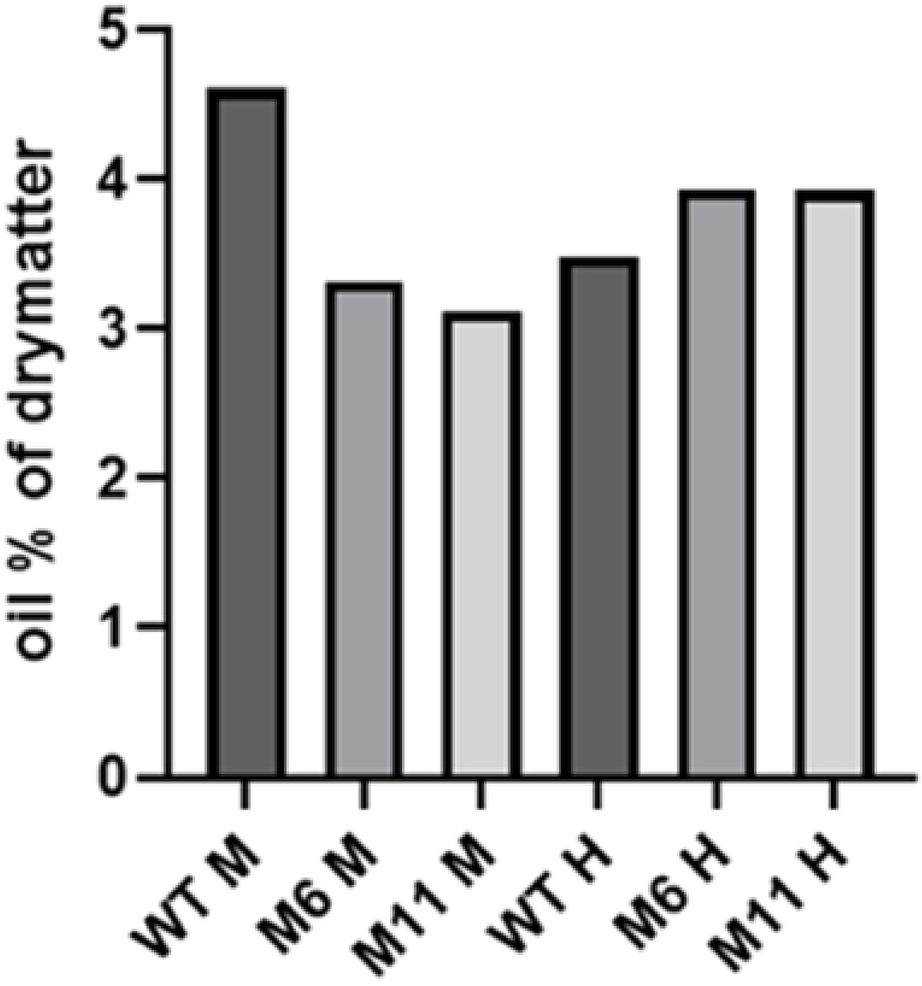

Lipid content in *C. vulgaris* WT and the two mutants M6 and M11 biomass.

Due to an insufficient amount of biomass only a single lipid measurement was obtained for each sample, hence the results should only be regarded as indicative.

M: mixotrophic,
H: heterotrophic cultivation.
